# ScReNI: Single-cell Regulatory Network Inference Through Integrating scRNA-seq and scATAC-seq Data

**DOI:** 10.1093/gpbjnl/qzaf060

**Published:** 2025-07-01

**Authors:** Xueli Xu, Yanran Liang, Miaoxiu Tang, Jiongliang Wang, Xi Wang, Yixue Li, Jie Wang

**Affiliations:** Center for Biomedical Digital Science, Guangzhou Institutes of Biomedicine and Health, Chinese Academy of Sciences, Guangzhou 510530, China; Center for Biomedical Digital Science, Guangzhou Institutes of Biomedicine and Health, Chinese Academy of Sciences, Guangzhou 510530, China; University of Chinese Academy of Sciences, Beijing 100049, China; Center for Biomedical Digital Science, Guangzhou Institutes of Biomedicine and Health, Chinese Academy of Sciences, Guangzhou 510530, China; University of Chinese Academy of Sciences, Beijing 100049, China; Center for Biomedical Digital Science, Guangzhou Institutes of Biomedicine and Health, Chinese Academy of Sciences, Guangzhou 510530, China; University of Chinese Academy of Sciences, Beijing 100049, China; Center for Biomedical Digital Science, Guangzhou Institutes of Biomedicine and Health, Chinese Academy of Sciences, Guangzhou 510530, China; Guangzhou National Laboratory, Guangzhou 510005, China; Key Laboratory of Systems Health Science of Zhejiang Province, School of Life Science, Hangzhou Institute for Advanced Study, University of Chinese Academy of Sciences, Hangzhou 310024, China; Center for Biomedical Digital Science, Guangzhou Institutes of Biomedicine and Health, Chinese Academy of Sciences, Guangzhou 510530, China; University of Chinese Academy of Sciences, Beijing 100049, China; Guangdong Provincial Key Laboratory of Biocomputing, Guangzhou Institutes of Biomedicine and Health, Chinese Academy of Sciences, Guangzhou 510530, China; China-New Zealand Joint Laboratory on Biomedicine and Health, Guangzhou 510530, China

**Keywords:** Single-cell multi-omics, Nearest neighbor, Cell-specific regulatory network, Network-based cell clustering, Cell-enriched regulator

## Abstract

Each cell possesses a unique gene regulatory network. However, limited methods exist for inferring cell-specific regulatory networks, particularly through the integration of single-cell RNA sequencing (scRNA-seq) and single-cell assay for transposase-accessible chromatin using sequencing (scATAC-seq) data. Herein, we develop a novel algorithm, named single-cell regulatory network inference (ScReNI), for inferring gene regulatory networks at the single-cell level. In ScReNI, the nearest neighbors algorithm is utilized to establish the neighboring cells for each cell, where nonlinear regulatory relationships between gene expression and chromatin accessibility are inferred through a modified random forest. ScReNI is designed to analyze both paired and unpaired datasets for scRNA-seq and scATAC-seq. ScReNI demonstrates more accurate regulatory relationships and outperforms existing cell-specific network inference methods in network-based cell clustering. ScReNI also shows superior performance in inferring cell type-specific regulatory networks through integrating gene expression and chromatin accessibility. Importantly, ScReNI offers the unique function of identifying cell-enriched regulators based on each cell-specific network. Overall, ScReNI facilitates the inference of cell-specific regulatory networks and cell-enriched regulators, providing insights into single-cell regulatory mechanisms of diverse biological processes. ScReNI is available at https://github.com/Xuxl2020/ScReNI.

## Graphical abstract

**Figure:**
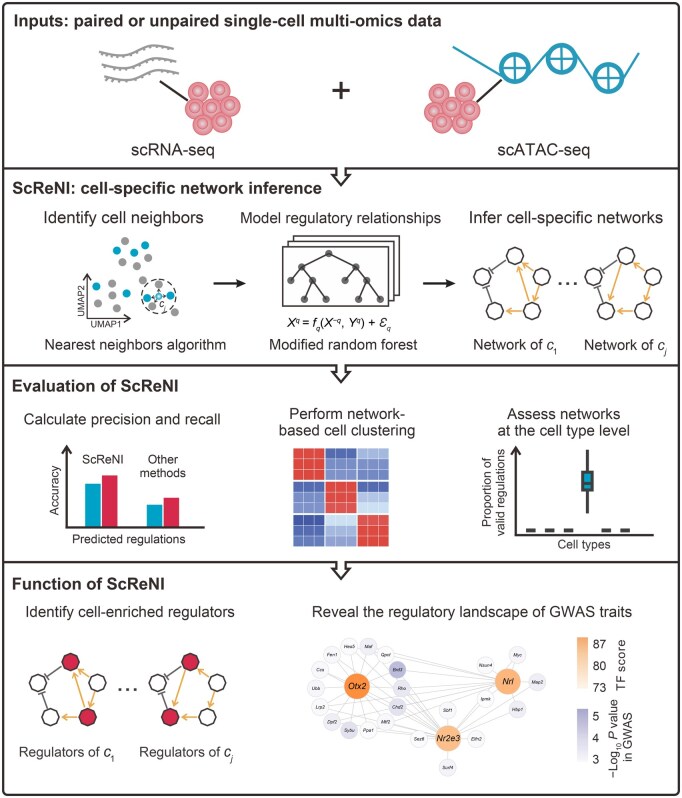

## Introduction

Transcriptional regulatory networks are essential for cells to determine which genes should be transcribed [1]. Typically, *trans*-regulators [*e.g.*, transcription factors (TFs)] bind to *cis*-regulatory elements to regulate gene transcription. Gene transcription levels can be globally measured using RNA sequencing (RNA-seq) technology [2]. The genomic locations of *cis*-regulatory elements, which are commonly present in open chromatin regions, can be detected using the assay for transposase-accessible chromatin using sequencing (ATAC-seq) [3]. The revolution of single-cell sequencing technology has led to the advent of single-cell RNA-seq (scRNA-seq) and single-cell ATAC-seq (scATAC-seq) [4]. These cutting-edge technologies enable the measurement of gene expression and chromatin accessibility at the single-cell level, either in different batches of cells or simultaneously within the same cells, resulting in either unpaired or paired datasets for scRNA-seq and scATAC-seq, respectively [5]. These single-cell sequencing data offer unprecedented opportunities for the accurate inference of regulatory networks [1].

Regulatory network analysis has become an indispensable tool for uncovering the underlying regulatory mechanisms and key regulatory factors that govern a myriad of biological processes [6–8]. By leveraging bulk RNA-seq data or scRNA-seq data, algorithms such as gene network inference with ensemble of trees (GENIE3), partial information decomposition and context (PIDC), and single-cell regulatory network inference and clustering (SCENIC) have been employed to infer cell type-specific regulatory networks [9–11]. Several methods like SCENIC+, integrated regulatory network analysis (IReNA), DeepTFni, single-cell multi-task network inference (scMTNI), and single-cell trajectory reconstruction, exploration and mapping of single-cell data (STREAM) have emerged, offering enhanced capabilities to infer cell type-specific regulatory networks by integrating scRNA-seq and scATAC-seq data [12–16]. Notably, individual cells, even within the same cell type, display distinct regulatory networks due to their heterogeneous profiles of gene expression and chromatin accessibility. This feature highlights the importance of inferring cell-specific regulatory networks, which are essential for uncovering the regulatory heterogeneity and key regulators at the single-cell level.

Currently, very few methods have been developed to infer cell-specific networks, primarily tailored for scRNA-seq data. For instance, cell-specific network (CSN) is designed to infer networks for each individual cell using scRNA-seq data [17]. It calculates binary values through a statistical measurement to quantify the dependencies between any two genes, thereby indicating gene regulatory relationships. The linear interpolation to obtain network estimates for single samples (LIONESS) has been used to infer networks through extracting sample-specific regulatory networks from population-level networks [18]. A deep learning approach, scGeneRAI, employs the layer-wise relevance propagation to infer gene regulatory networks of individual cells from scRNA-seq data [19]. The cell-specific gene regulatory network inference (CeSpGRN) reconstructs cell-specific regulatory networks from scRNA-seq data [20]. When paired scATAC-seq data are available, CeSpGRN only uses chromatin accessibility to narrow down the regulatory relationships inferred from scRNA-seq data, but does not establish the essential relationships between scRNA-seq and scATAC-seq data [20]. The lifelong neural network for gene regulation (LINGER) infers cell-specific gene regulatory networks from paired scRNA-seq and scATAC-seq data by using external atlas-scale data [21]. Nevertheless, the method of integrating scRNA-seq and scATAC-seq data to infer cell-specific regulatory networks is still quite limited, particularly when dealing with unpaired scRNA-seq and scATAC-seq data.

In this study, we developed a new computational method named single-cell regulatory network inference (ScReNI), which constructs cell-specific regulatory networks from scRNA-seq and scATAC-seq data in an unpaired or paired manner. Specifically, ScReNI initially integrates unpaired scRNA-seq and scATAC-seq datasets through aligning them in a shared analytical space. It then establishes the association between genes and peaks across all cells through genomic proximity and motif analysis. Finally, ScReNI uses *k*-nearest neighbors and random forest algorithms to infer gene regulatory relationships for individual cells by modeling the nonlinear relationships between scRNA-seq and scATAC-seq data. This methodology, called weighted-nearest neighbor ScReNI (wScReNI), is primarily utilized for analyzing scRNA-seq and scATAC-seq data. The aforementioned method can also be used to analyze scRNA-seq data alone for cell-specific network inference, named *k*-nearest neighbor ScReNI (kScReNI), using the *k*-nearest neighbors algorithm to identify the neighboring cells of each cell. To assess the performance of ScReNI compared to existing methods, we also utilized scRNA-seq data alone to infer cell-specific networks. Through validation using unpaired and paired single-cell sequencing datasets, we demonstrated that a higher fraction of regulatory relationships inferred by ScReNI are detected by chromatin immunoprecipitation sequencing (ChIP-seq) data. ScReNI shows superior performance in network-based cell clustering when compared to existing cell-specific network inference methods. Importantly, ScReNI enables the identification of cell-enriched regulators based on cell-specific regulatory networks.

## Method

### Overview of ScReNI

**Figure 1:**
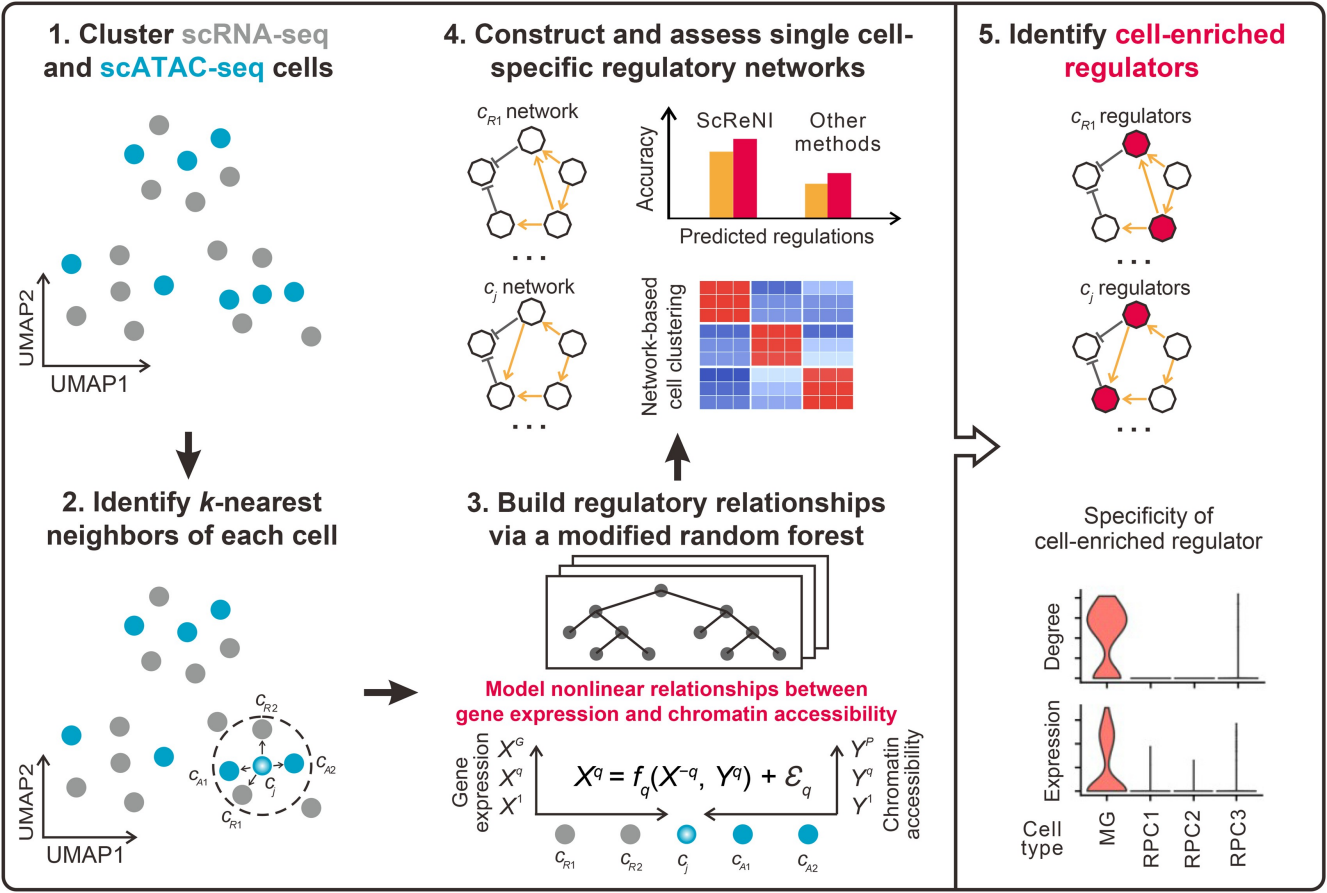
Overview of ScReNI ScReNI contains five major parts. First, cells measured by scRNA-seq and scATAC-seq are clustered together. Second, neighbors for each cell are identified using the *k*-nearest neighbors algorithm. Third, the weights of TFs regulating TGs are calculated using random forest which models nonlinear relationships between scRNA-seq and scATAC-seq data. Fourth, ScReNI is validated by assessing cell-specific regulatory networks. Fifth, cell-enriched regulators are identified based on each cell-specific network. ScReNI, single-cell regulatory network inference; scRNA-seq, single-cell RNA sequencing; scATAC-seq, single-cell assay for transposase-accessible chromatin using sequencing; UMAP, uniform manifold approximation and projection; MG, Müller glial; RPC, retinal progenitor cell; TF, transcription factor; TG, target gene.

ScReNI is developed to infer cell-specific regulatory networks from unpaired (or paired) scRNA-seq and scATAC-seq data (**Figure 1**, Figure S1A). The count matrices of gene expression and chromatin accessibility are independently generated from scRNA-seq and scATAC-seq data, respectively. These matrices are then used to predict cell-specific regulatory networks through four key steps: clustering of cells measured by scRNA-seq and scATAC-seq, identification of *k*-nearest neighbors for each cell, inference of gene regulatory relationships via the random forest, and reconstruction and evaluation of cell-specific regulatory networks. After performing these four steps, regulators enriched in each cell will be identified through statistically analyzing cell-specific regulatory networks.

In ScReNI, we employ the weighted-nearest neighbor analysis from Seurat software [22] to integrate paired scRNA-seq and scATAC-seq data. This analytical strategy allows us to identify the nearest neighbors for each cell and calculate the relative utility of each data type from scRNA-seq and scATAC-seq. In contrast, for the integration of unpaired scRNA-seq and scATAC-seq data, we apply the Seurat anchoring procedure to identify anchors between the two data types, followed by cell clustering using Harmony [23,24]. Subsequently, we identify the neighboring cells of each cell based on single-cell clustering and the weighted-nearest neighbors algorithm. The cell itself and its neighboring cells are utilized to infer the regulatory network of the single cell.

The determination of associations between genes and peaks in ScReNI contains a two-step process (Figure S1B). First, genes are associated with peaks which are located within 250 kb upstream and 250 kb downstream of transcription start sites of the gene. This restriction is strategically implemented to minimize the risk of generating false predictions. Using the “findOverlaps” function from the R package “GenomicRanges”, we ascertain which genes overlapped with region-specific peaks. Second, the correlation between genes and peaks across cells is established by calculating an absolute value of the Spearman correlation coefficient. Then, the “matchMotifs” function from the R package “motifmatchr” is employed to pinpoint potential binding sites of TFs within the genomic regions. Genes are considered associated with peaks if the Spearman correlation coefficient between the gene and peak is greater than 0.1.

ScReNI applies gene–peak associations to a group of cells comprising the cell of interest and its *k*-nearest neighbors. Utilizing random forest, ScReNI builds ensembles of regression models to integrate gene expression from scRNA-seq data and chromatin accessibility of gene-associated peaks from scATAC-seq data. The important scores calculated by random forest thereby are used as the regulatory weights of TFs on their target genes (TGs). For each gene, the algorithm evaluates the influence of all other genes, including peak-associated TFs. The weights are then applied to reconstruct the regulatory network for an individual cell. wScReNI is primarily utilized for analyzing scRNA-seq and scATAC-seq data. kScReNI is used to analyze scRNA-seq data alone for cell-specific network inference.

To benchmark the performance of ScReNI against existing methods, we reconstructed cell-specific networks using CSN, LIONESS, CeSpGRN, scGeneRAI, and LINGER. These methods infer cell-specific regulatory networks solely based on transcriptomic data. For this purpose, we employed two types of publicly available datasets: one consisting of unpaired scRNA-seq and scATAC-seq data from retinal development, and the other containing paired scRNA-seq and scATAC-seq data from peripheral blood mononuclear cells (PBMCs) of a healthy donor. We utilized these datasets to validate the inferred cell-specific regulatory networks. Subsequently, two downstream analyses were conducted. First, regulators specifically enriched in individual cells were identified based on the reconstructed cell-specific regulatory networks. Second, disease-related or trait-related cell types, TFs, and regulatory networks were analyzed through integrating cell-specific regulatory networks with genome-wide association study (GWAS) data, following the approach used in LINGER [21].

### Datasets used to illustrate ScReNI

Two types of high-quality scRNA-seq and scATAC-seq data were measured separately in unpaired and paired manners to assess the performance of the ScReNI method.

The unpaired scRNA-seq and scATAC-seq data were publicly available from the study of retinal development in mice, which can be accessed at Gene Expression Omnibus (GEO: GSE181251) [25]. The original research reported 13 major retinal cell types. For this study, we focused on three subtypes of retinal progenitor cells (RPCs), designated as RPC1, RPC2, and RPC3, as well as Müller glial (MG) cells, utilizing their original cell type annotations.

The paired scRNA-seq and scATAC-seq data were obtained from PBMCs of a healthy donor which were obtained by 10X Genomics (https://www.10xgenomics.com/datasets/pbmc-from-a-healthy-donor-no-cell-sorting-10-k-1-standard-2-0-0). The libraries for paired gene expression and chromatin accessibility were generated from the isolated nuclei as described in the Chromium Next GEM Single Cell Multiome ATAC + Gene Expression User Guide (CG000338 Rev A) and sequenced on Illumina NovaSeq 6000 v1 Kit (Forward Strand Dual-Indexed Workflow, Illumina, San Diego, CA). For this research, we selected single-cell sequencing data from CD14 monocytes, CD16 monocytes, CD4 naive cells, CD8 naive cells, conventional dendritic cells (cDCs), memory B cells, natural killer (NK) cells, and regulatory T (Treg) cells.

### Cell clustering through integrating scRNA-seq and scATAC-seq data

In ScReNI, the weighted-nearest neighbor analysis from Seurat software [22] is utilized to integrate paired scRNA-seq and scATAC-seq data. For unpaired scRNA-seq and scATAC-seq data, we initiate our analysis by generating an estimate of the transcriptional activity of each gene. This is accomplished by quantifying ATAC-seq counts within the gene body and its 2-kb upstream region, using the GeneActivity function in the Signac package [26]. The resulting gene activity scores, derived from the scATAC-seq data, serve as inputs for canonical correlation analysis. Following this, we apply the Seurat v5 anchoring procedure to identify anchors between the scRNA-seq and scATAC-seq data [27]. Once anchors are identified, we facilitate the transfer of annotations from the scRNA-seq dataset onto the scATAC-seq cells. Following the anchoring, we proceed with cell clustering using Harmony [24].

### Identification of the nearest neighbors for each cell

For each cell, the weighted nearest neighbor procedure implemented in the R package Seurat (v5) [22] learns a set of modality weights. The identification of the nearest neighbors of the single cell is achieved through a multi-step process. Initially, a Seurat object is constructed from scRNA-seq data. Subsequently, this is complemented by integrating scATAC-seq data as an additional assay within the same Seurat object. Both assays are then independently subjected to standard pre-processing and dimensionality reduction which are specific to data types. Furthermore, a weighted nearest neighbor graph is computed, which effectively combines the information from both scRNA-seq and scATAC-seq modalities. This graph is essential for subsequent uniform manifold approximation and projection (UMAP) visualization and clustering, providing a comprehensive framework for identifying the nearest neighbors.

### Regulatory network inference in ScReNI

ScReNI is a new tool designed to infer cell-specific regulatory networks through integrating scRNA-seq and scATAC-seq data, accommodating both unpaired and paired datasets. ScReNI operates on a dataset comprising N samples (cells) and G+P features (G genes and P peaks).

The main hypothesis is that the expression of a TG is influenced by the TFs and the peaks associated with the TG. Let $X^{-q}$ represent the vector of expression values for all genes except gene q, represented as:


(1) $$X^{-q}={\left(X^{1},X^{2},\ldots ,X^{\left(q-1\right)},X^{\left(q+1\right)},\cdots ,X^{G}\right)}^{T}$$

We propose that the expression level $X^{q}$ of the TG q is regulated by the TFs present in $X^{-q}$ and the peaks in $Y^{q}$. This regulatory relationship is formalized in the model:


(2) $$X^{q}=f_{q}(X^{-q},Y^{q})+\varepsilon_{q}$$

where $\varepsilon_{q}$ represents random noise with a mean of zero, and the function $f_{q}$ represents the important scores encapsulating the regulatory effects of the TFs in $X^{-q}$ on gene q, considering the peaks in $Y^{q}$ that are associated with gene q.

ScReNI predicts regulatory links pointing to gene q by learning the function $f_{q}$. Within the ScReNI framework, the regulatory function $f_{q}$ is modeled using a random forest method, employing the default parameters. The random forest method returns the important scores of all TFs in $X^{-q}$ and peaks in $Y^{q}$ using IncNodePurity and IncMSE metrics.

### Determination of gene regulatory relationships

The regulatory weight $w_{i,j}$ between genes i and j is determined by aggregating the importance scores of gene j and the associated peaks of gene i, which are calculated from the random forest. The regulatory weight $w_{i,j}$ between genes i and j is calculated using the following formula:


(3) $$w_{i,j}=z_{i,j}+\sum_{l}p_{i,l}I_{i,j}$$

(4) $$I_{i,j}=\{\begin{array}{l} 1,\;\mathrm{if}\;\mathrm{gene}\;j\;\mathrm{is}\;a\;\mathrm{TF}\;\mathrm{and}\;\mathrm{binds}\;\mathrm{to}\;\mathrm{peak}\;l\;\mathrm{of}\;\mathrm{gene}\;i\;\\ 0,\;\mathrm{otherwise}\; \end{array}$$

where $z_{i,j}$ denotes the importance score of gene j regulating TG i, $p_{i,l}$ represents the importance score of the peak l associated with gene i, and $I_{i,j}$ refers to an indicator of whether gene j is related to the peaks associated with gene i.

### Construction of cell-specific regulatory networks

For a given single cell c (c=1, 2, ⋯, N), the *k*-nearest neighboring cells are identified through a weighted nearest neighbor analysis. Cell *c* and its neighboring cells are then used as inputs of Equation 2 to infer the regulatory network of the single cell *c*. The obtained cell-specific regulatory networks are directed from regulators to TGs. The regulatory weights between genes represent the regulatory strengths.

### Inference and evaluation of cell type-specific regulatory networks

For cell type-specific regulatory network inference, we evaluated the performance of wScReNI, LINGER, DeepTFni, STREAM, and scMTNI [12–16]. Paired scRNA-seq and scATAC-seq data from PBMCs were used for comparing wScReNI with LINGER, DeepTFni, and STREAM. Since scMTNI requires lineage information, we compared wScReNI and scMTNI using data from retinal development. For wScReNI and LINGER, which enable to predict cell-specific regulatory networks, cell type-specific regulatory networks are inferred either by averaging cell-specific regulatory networks within the same cell type (indirect approach) or directly using single-cell sequencing data from the same cell type as inputs of ScReNI (direct approach). In the direct approach, the number of neighbors (*k*) in the nearest neighbors algorithm is set to equal the number of cells within the same cell type. The indirect approach is used to compare wScReNI and LINGER. The direct cell type-specific regulatory networks inferred by wScReNI are used for comparison with DeepTFni, STREAM, and scMTNI.

### Calculation of network similarity

For each gene regulatory network, regulation pairs are ranked according to their weights. Based on the top 3000 regulation pairs, we calculated the number of regulatory relationships shared by two different cells to measure the similarity between the corresponding networks of the cells.

### Baseline models

CSN, LIONESS, CeSpGRN, scGeneRAI, and LINGER were used as the baseline models to infer cell-specific regulatory networks [21]. LINGER, DeepTFni, scMTNI, and STREAM were used as the baseline models to infer cell type-specific regulatory networks [12–16]. All default settings were used. CSN analyzes scRNA-seq data at the single-cell level. It employs a statistical model to identify gene–gene associations based on their joint and marginal probabilities. LIONESS estimates individual-specific regulatory networks from gene expression data. The method is based on the principle that the presence or absence of a single sample can cause a slight change in an overall network model. This change is utilized to assess the impact of a sample on the aggregate network, which in turn helps to determine a sample-specific network. CeSpGRN employs a Gaussian weighted kernel to model the gene expression from scRNA-seq data and learn the gene regulatory network of a given cell. The kernel is constructed from the similarity of gene expression or spatial locations between cells. When paired scATAC-seq data are available, CeSpGRN connects the TFs to their corresponding TGs based on the chromatin accessibility measured by scATAC-seq, and then narrows down the regulatory relationships between TFs and TGs inferred from the scRNA-seq data. scGeneRAI uses an explicable artificial intelligence known as layer-wise relevance propagation to infer single-cell gene regulatory networks from scRNA-seq data. LINGER integrates large-scale external data and TF-response element (RE) motif matching knowledge via manifold regularization, enabling the inference of population, cell type-specific, and cell-specific gene regulatory networks. Each gene regulatory network includes three interactions: *trans*-regulation (TF–TG), *cis*-regulation (RE–TG), and TF binding (TF–RE). DeepTFni infers TF regulatory networks with variational graph auto-encoder using scATAC-seq data. Binary values indicate whether there is an interaction or not between two TFs. scMTNI is a multi-task learning framework to infer the gene regulatory network for each cell type on a lineage from scRNA-seq and scATAC-seq data. STREAM is a computational framework for inferring enhancer-driven gene regulatory networks from paired scRNA-seq and scATAC-seq data, using the Steiner Forest Problem model and submodular optimization.

### Assessment of the runtime and memory usage

Computational analyses were executed on a Linux Ubuntu system, equipped with a 104-core Intel^R^ Xeon^R^ Gold 5320 central processing unit (CPU) operating at 2.20 GHz and 1.0 terabyte of random access memory (RAM). The same numbers of cells, highly variable genes, and highly variable peaks were used as the inputs to benchmark different methods. The runtime was calculated using the R function “system.time”. The memory usage was monitored by the R function “gc”.

### Evaluation of the precision and recall on predicting regulatory networks

ChIP-Atlas was used to assess the inferred regulatory relationships [28,29]. We introduced the ChIP-Atlas dataset, a widely used gold standard network dataset that contains regulatory interactions detected by high-throughput sequencing experiments, such as ChIP-seq and ATAC-seq. This dataset serves as a valuable reference for evaluating the performance of different network inference methods. Precision and recall serve as metrics for evaluating the performance of different cell-specific or cell type-specific regulatory network inference methods. The results were visualized using histograms or boxplots for each regulatory network inference method. The thresholds were chosen to prioritize different numbers of top regulation pairs which were ranked based on regulatory weights.

### Clustering of single cells based on cell-specific networks

For network-based cell clustering, we calculated gene degrees derived from cell-specific networks. The “hclust” function in R was used for hierarchical clustering. To evaluate the influence of the number of pairwise relationships between TFs and TGs, we applied diverse thresholds to the ranked regulatory weights. Specifically, we selected the top 500, 800, 1000, and 3000 regulation pairs. To quantitatively measure the similarity between the resulting clustering and the known ground truth of cell types, we employed the adjusted rand index (ARI) as in the previous study [30]. This metric is implemented in the “randIndex” function from the R package “flexclust”, providing a robust statistical measure for evaluating the accuracy of clustering methods [31].

### Identification of cell-enriched regulators

To investigate the trajectory and pseudotime of retinal cells using scRNA-seq data of mouse retinal development, we transferred the data to Monocle 3 using SeuratWrappers [32]. Cells were clustered via UMAP reduction, and the principal graph was determined using the “learn_graph” function, with parameters set to euclidean_distance_ratio = 5, geodesic_distance_ratio = 2, and minimal_branch_len = 1000. Other parameters were kept at their default settings. Any cell in RPC1 was set as the root when running “order_cells”. These data were then added back to Seurat as metadata for further analysis.

Considering the sparsity of scRNA-seq data, we employed the smoothed expression profiles for gene co-expression analysis. Pseudotime was divided into 50 equal intervals. Highly variable genes and expressed TFs were grouped into distinct modules using K-means clustering on the smoothed expression profiles. The optimal number of modules was determined by calculating the average silhouette score for K-means clustering, using the R package “cluster”. For each module, we conducted functional enrichment analysis using clusterProfiler, leveraging Gene Ontology (GO) and Kyoto Encyclopedia of Genes and Genomes (KEGG) databases [33].

Based on modules of genes, the inferred regulatory network from each cell was modularized. The hypergeometric test with false discovery rate multiple hypotheses test correction was applied to calculate and calibrate the probability *P* that a given TF significantly regulates a specific gene module. TFs identified as significantly enriched regulators of gene modules were determined using a false discovery rate threshold of 0.01.

### Statistical analysis

All statistical analyses were conducted using R (v.4.3.3) software. Spearman rank correlation coefficients were used to examine the association between network degrees and expression levels of each cell-enriched regulator across all four cell types. Kruskal–Wallis test with false discovery rate correction was used to compare the statistical differences in network degrees, expression levels, and regulatory activities of cell-enriched regulators among four cell types (MG, RPC1, RPC2, and RPC3). The two-tailed *t*-test was performed to statistically compare the mean number of TFs negatively and positively regulating the TG. Statistical significance was set at *P* < 0.05.

## Results

### ScReNI constructs cell-specific regulatory networks using unpaired scRNA-seq and scATAC-seq data

The unpaired scRNA-seq and scATAC-seq data were obtained from the previous study on mouse retinal development [25]. We used scRNA-seq data and scATAC-seq data from age-matched nine time points between two sequencing types. The original study reported 13 major retinal cell types. To illustrate ScReNI, we only focused on three subtypes of RPCs (RPC1, RPC2, and RPC3), as well as MG cells. The original annotation of cell types was used as the ground truth in downstream analysis.

**Figure 2:**
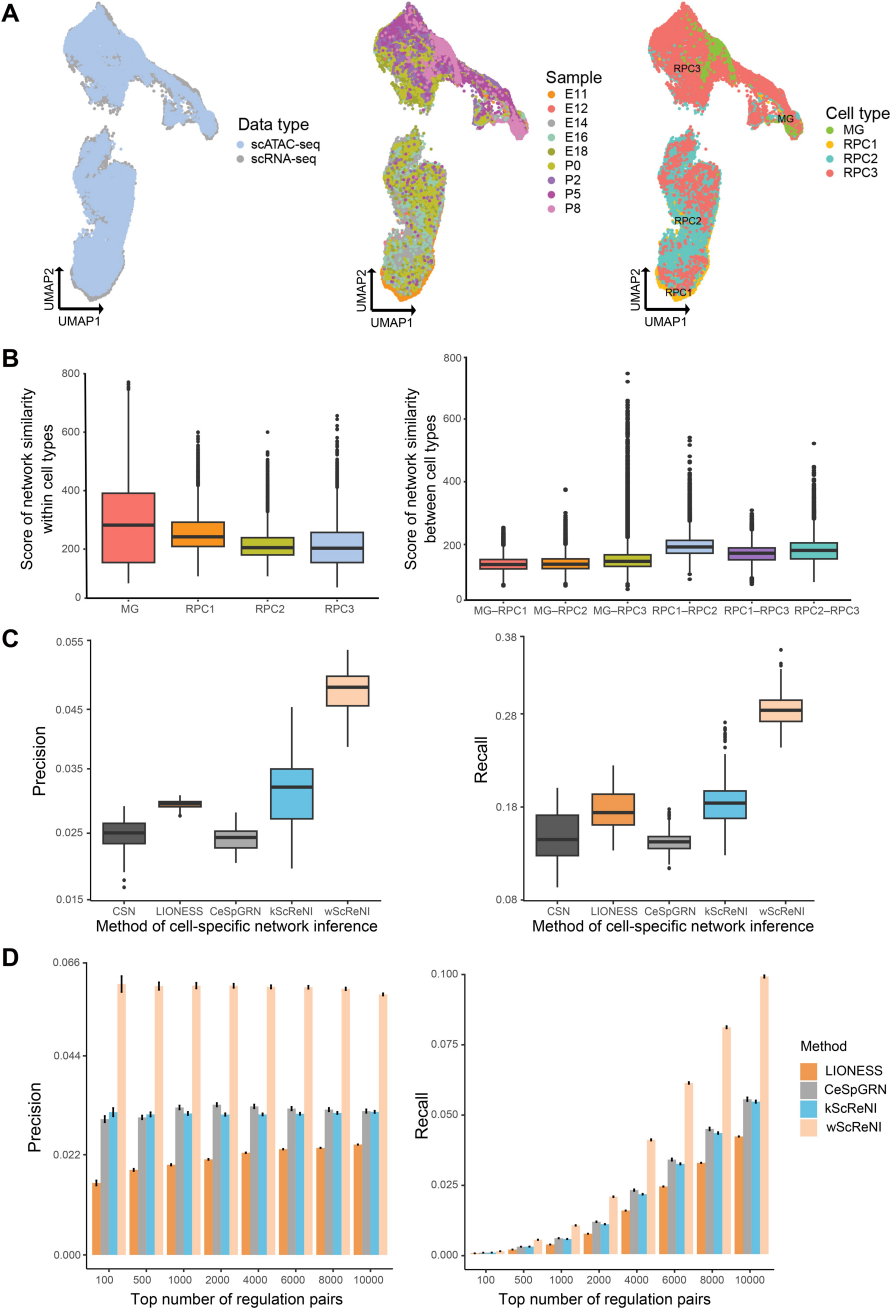
Cell-specific network analysis of unpaired scRNA-seq and scATAC-seq data from retinal development **A**. UMAP visualization of different data types, samples, and cell types following multi-omics integration. **B**. Similarities of gene regulatory networks separately within cell types and between cell types. **C**. Precision and recall of single-cell regulatory relationships for different network inference methods using the same number of regulation pairs in CSN. **D**. Precision and recall of single-cell regulatory relationships for four inference methods using different top numbers of regulation pairs. CSN, cell-specific network; LIONESS, linear interpolation to obtain network estimates for single samples; CeSpGRN, cell-specific gene regulatory network inference; wScReNI, weighted-nearest neighbor ScReNI; kScReNI, *k*-nearest neighbor ScReNI.

The scRNA-seq dataset encompassed 7853 RPC1, 16,645 RPC2, 22,943 RPC3, and 936 MG cells, complemented by the scATAC-seq dataset with 6049 RPC1, 10,464 RPC2, 11,912 RPC3, and 768 MG cells. Integration of these datasets was executed using Seurat anchor-based integration and Harmony [22,24]. For the single-cell clustering, we used the top 2000 highly variable genes and the top 10,000 highly variable peaks. The integrative analysis of scRNA-seq and scATAC-seq data showed a substantial overlap between the same cell types identified by two sequencing types (**Figure 2**A). The clustering results also delineated the developmental trajectory of MGs from RPCs, which was consistent with the original report [25]. According to cell clustering, we determined the neighboring cells for each cell using the weighted-nearest neighbors algorithm. Subsequently, we randomly chose 100 cells from each type of RPCs and MGs as a representative subset of 400 cells for cell-specific network reconstruction. For each cell and its neighbors, gene regulatory networks were inferred using wScReNI. Then, the regulation pairs in regulatory networks were ranked according to the weights computed by wScReNI. Based on the top 3000 regulation pairs, we analyzed the similarities of gene regulatory networks separately within cell types and between cell types (Figure 2B). The median network similarities within the MG, RPC1, RPC2, and RPC3 were 282, 242, 205, and 203, respectively. The median network similarity between RPC1 and RPC2 was 192, which is the highest among all pairs of different cell types. The results indicate that each cell contains a specific and distinct regulatory network. Cells from the same cell type have more similar regulatory networks than those from different cell types.

### ScReNI shows superior performance on regulatory network inference

To assess the accuracy and reliability of the inferred regulatory relationships, we utilized the binding sites of TFs measured by ChIP-seq as the gold standard for gene regulatory relationships. The ChIP-seq data were from the ChIP-Atlas database [34]. Following this, we calculated the precision and recall of cell-specific regulatory networks inferred by ScReNI, CSN, LIONESS, and CeSpGRN.

We first used the top 500 highly variable genes and the top 10,000 highly variable peaks for network inference. In network inference, wScReNI, kScReNI, LIONESS, and CeSpGRN calculated the continuous values for regulatory weights. However, the CSN method produced binary values for regulatory weights representing whether the gene is regulated or not. For each cell-specific regulatory network from retinal development, the regulation pairs were ranked based on their weights. To perform a fair comparison across different cell-specific network inference methods, we selected the same number of regulation pairs predicted by CSN. The results showed that both wScReNI and kScReNI had higher precision and recall than other methods (Figure 2C). wScReNI showed the best performance among all methods. Specifically, the mean precision values for CSN, LIONESS, CeSpGRN, kScReNI, and wScReNI were 0.024, 0.029, 0.023, 0.031, and 0.047, respectively. The mean recall values for CSN, LIONESS, CeSpGRN, kScReNI, and wScReNI were 0.149, 0.179, 0.144, 0.185, and 0.288, respectively. We also calculated the precision and recall values of wScReNI, kScReNI, CeSpGRN, and LIONESS using different top numbers of regulation pairs (Figure 2D). Remarkably, wScReNI consistently outperformed other methods across all groups of cell-specific regulatory networks.

To confirm the aforementioned results, we also used the top 2000 highly variable genes and the top 10,000 highly variable peaks to infer cell-specific regulatory networks. The best performance was also observed in wScReNI regardless of the number of regulation pairs used in network inference (Figure S2A and B). These results indicate that ScReNI, especially wScReNI, has a remarkable performance on the accurate inference of cell-specific regulatory networks.

### ScReNI outperforms current methods in network-based cell clustering

**Figure 3:**
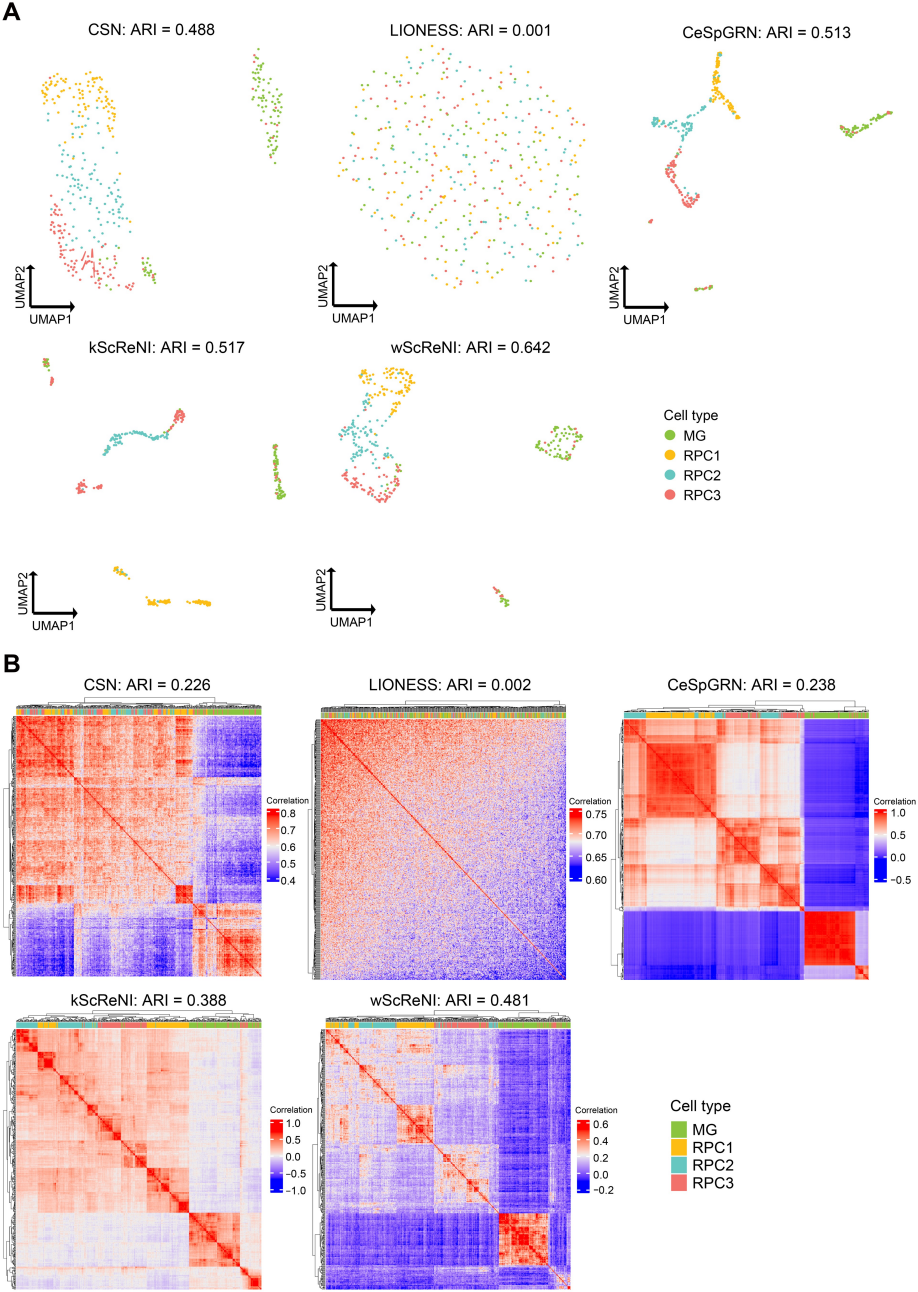
Network-based cell clustering of unpaired scRNA-seq and scATAC-seq data from retinal development **A**. UMAP of single cells using top 500 regulation pairs. **B**. Hierarchical clustering of single cells using top 500 regulation pairs. ARI, adjusted rand index.

To perform cell clustering analysis based on cell-specific networks, we computed the outdegrees of genes in each cell-specific network and obtained the matrix of gene outdegrees across all cells. According to gene degrees from the top 500 regulation pairs, single cells were clustered through Seurat and visualized using UMAP. Alternatively, single cells were clustered using hierarchical clustering and visualized using a heatmap. The clustering based on gene degrees of cell-specific networks from wScReNI showed the best separation of different cell types (**Figure 3**A). We calculated the ARI to measure the similarity between the originally reported cell types and those assigned from network-based clustering. The results showed that wScReNI and kScReNI had higher ARI values than other methods. Specifically, the ARI values of CSN, LIONESS, CeSpGRN, kScReNI, and wScReNI were 0.488, 0.001, 0.513, 0.517, and 0.642, respectively. Then, we performed hierarchical clustering and drew a heatmap using the top 500 regulation pairs. In wScReNI, the same cell types were more distinctly clustered together (Figure 3B). Notably, wScReNI and kScReNI exhibited higher ARI values compared to other methods. Specifically, the ARI values of CSN, LIONESS, CeSpGRN, kScReNI, and wScReNI were 0.226, 0.002, 0.238, 0.388, and 0.481, respectively. Network-based clustering analysis also showed that ScReNI had better performance in distinguishing different cell types, even sub-cell types of RPCs (RPC1, RPC2, and RPC3). To further validate the performance, we conducted single-cell clustering based on different top regulation pairs, such as 500, 800, 1000, and 3000 regulation pairs. The results consistently showed that ScReNI outperformed the other methods (Table S1). Additionally, we also conducted network-based single-cell clustering using 2000 highly variable genes and different numbers of top regulation pairs. A similar result was observed that wScReNI showed the best performance overall, except for a few cases that were comparable to those obtained by the CeSpGRN method (Table S1).

### ScReNI demonstrates superior performance in network inference using paired scRNA-seq and scATAC-seq data

To demonstrate the utility of ScReNI with paired scRNA-seq and scATAC-seq data, we employed publicly accessible single-cell multimodal datasets derived from PBMCs of a healthy donor, generated using the 10X Genomics platform. The dataset contains eight cell types, including 2812 CD14 monocytes, 514 CD16 monocytes, 1419 CD4 naive cells, 1410 CD8 naive cells, 198 cDC cells, 371 memory B cells, 468 NK cells, and 162 Treg cells. To construct cell-specific regulatory networks, we selected a representative subset of 400 cells, ensuring that each of the eight cell types contained 50 cells at random. The PBMC data were analyzed based on the top 500 highly variable genes and the top 10,000 highly variable peaks. Employing the weighted-nearest neighbors method, we performed single-cell clustering of scRNA-seq and scATAC-seq data, and then identified neighboring cells to infer the regulatory network for each cell.

**Figure 4:**
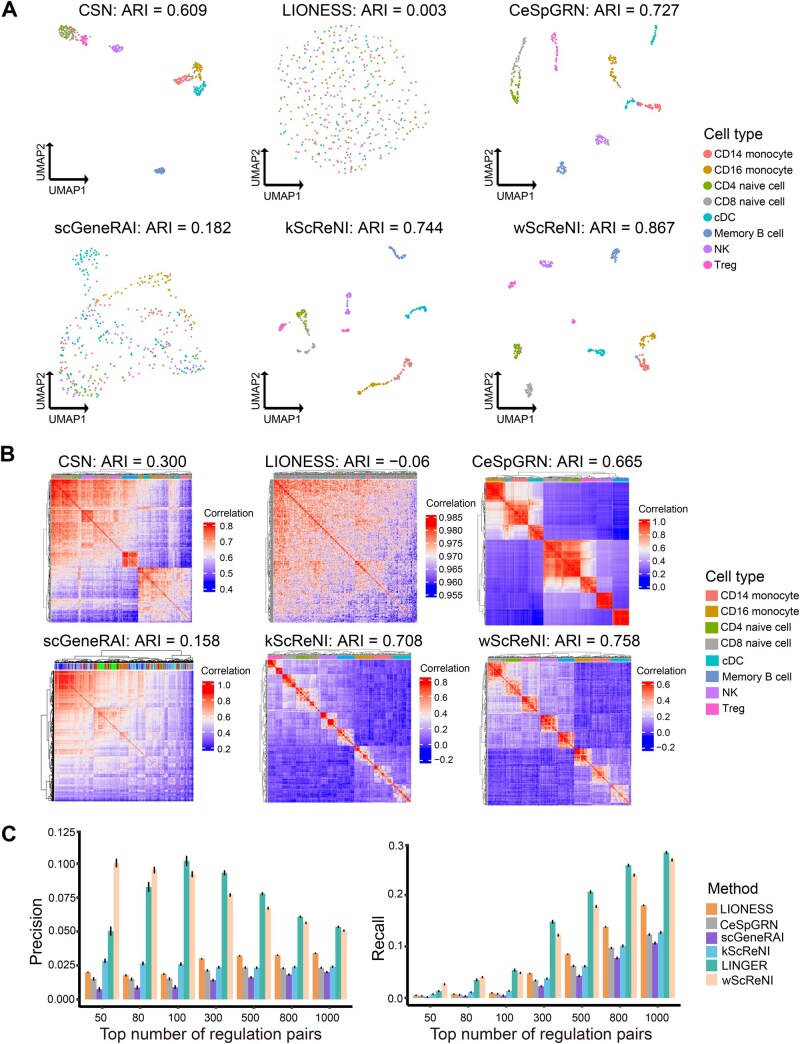
Network analysis of paired scRNA-seq and scATAC-seq data from PBMCs **A**. UMAP of single cells using top 500 regulation pairs. **B**. Hierarchical clustering of single cells using top 500 regulation pairs. **C**. Precision and recall of single-cell regulatory relationships for 500 highly variable genes using different top numbers of regulation pairs. PBMC, peripheral blood mononuclear cell; cDC, conventional dendritic cell; NK, natural killer; Treg, regulatory T cell; LINGER, lifelong neural network for gene regulation.

In the analysis of the PBMC dataset, network-based cell clustering was also applied to the gene outdegrees of the top 500 regulation pairs. UMAPs clearly illustrated that wScReNI provided better separation between different cell types compared to other methods (**Figure 4**A). This distinction was further corroborated by hierarchical clustering of the same gene degree matrix, which demonstrated distinct clustering patterns for various cell types within the wScReNI framework (Figure 4B). The ARI values were the highest in wScReNI, followed by kScReNI. Specifically, the ARI values of wScReNI were 0.867 and 0.758 for UMAPs and heatmaps, respectively. The results were highly consistent between the single-cell clustering and the ground truth of cell labels. The ARI values of CSN, LIONESS, scGeneRAI, CeSpGRN, and kScReNI were 0.609, 0.003, 0.182, 0.727, and 0.744 in UMAPs, respectively. In heatmaps, kScReNI yielded an ARI value of 0.708, whereas CSN, LIONESS, scGeneRAI, and CeSpGRN had ARI values not exceeding 0.670. We also chose different top numbers of regulation pairs (500, 800, 1000, and 3000) to calculate gene degrees and cluster single cells. The ARI values provided strong evidence that wScReNI had superior performance against other methods in single-cell clustering analysis (Table S2). Additionally, we also conducted single-cell clustering based on 2000 highly variable genes and different top regulation pairs. Similar results were observed that wScReNI presented a better performance (Table S2). The consistent performance across different datasets indicates the robustness and reliability of wScReNI for regulatory network inference.

Given the distinct strategy employed by LINGER to compute cell-specific regulatory networks from paired scRNA-seq and scATAC-seq data, we conducted a separate benchmark comparing LINGER, ScReNI, and other methods. Based on the aforementioned subset of 400 cells and 500 highly variable genes, LINGER identified 21 TFs and constructed cell-specific regulatory networks involving 348 TGs. To ensure a fair comparison, we also used LIONESS, CeSpGRN, scGeneRAI, kScReNI, and wScReNI to construct cell-specific gene regulatory networks between these 21 TFs and the corresponding 348 TGs, using different top regulation pairs. Although LINGER showed higher precision and recall rates than wScReNI when regulation pairs exceed 100, wScReNI consistently demonstrated the highest precision and recall rates for top 50 and 80 regulation pairs (Figure 4C). Using the same number of regulation pairs predicted by CSN, wScReNI and LINGER gave similar precision and recall, significantly higher than the other methods (Figure S2C). Furthermore, we compared the performance of network-based cell clustering using LINGER and wScReNI based on 21 TFs and the corresponding 348 TGs, using different top regulation pairs. The results showed that wScReNI had superior performance against LINGER in single-cell clustering analysis (Table S3). These findings underscore the superior performance of wScReNI in identifying reliable cell-specific regulatory relationships.

**Figure 5:**
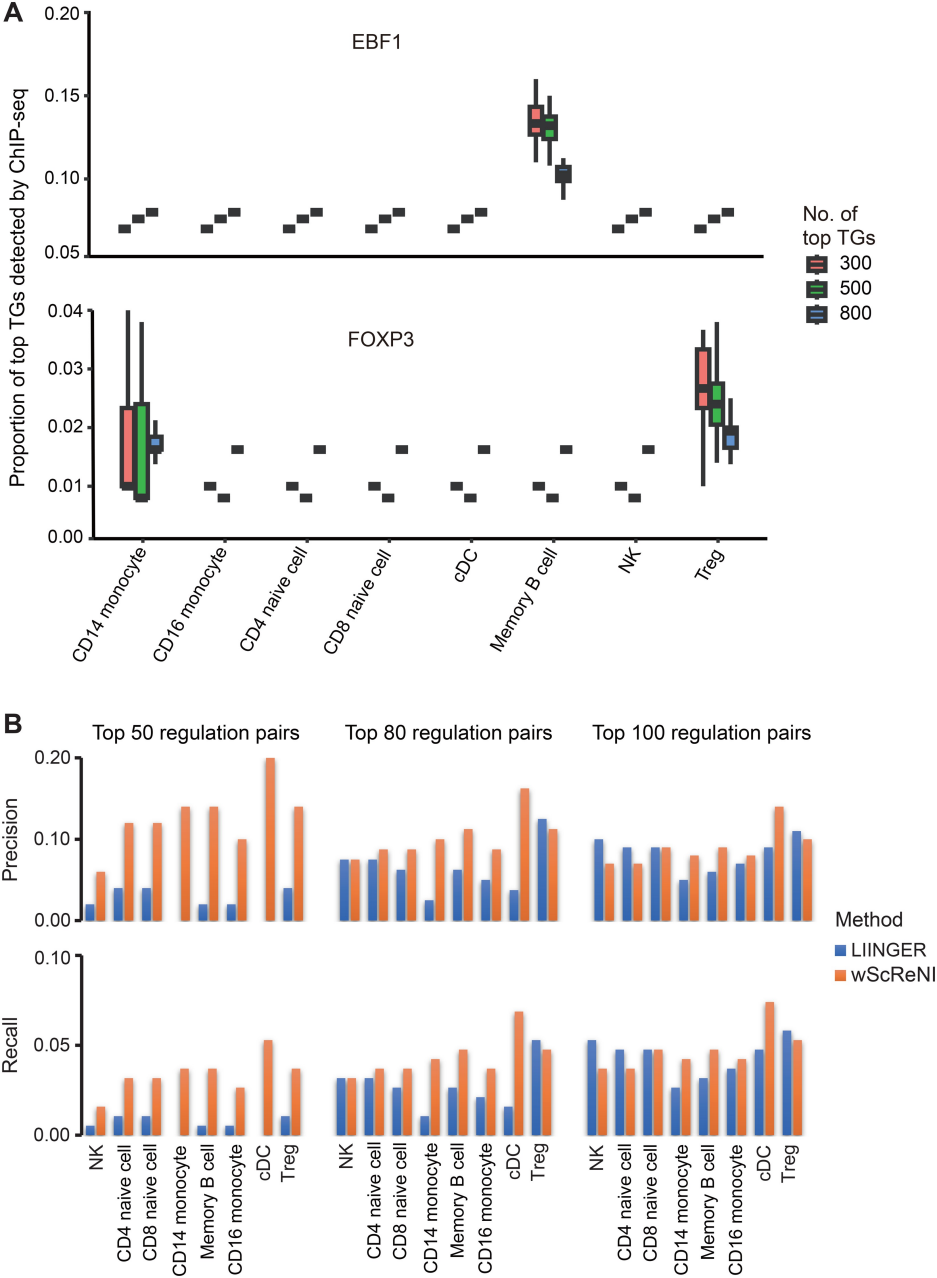
Performance of cell-specific regulatory networks inferred by ScReNI at the cell type level **A**. Proportions of the top 300, 500, and 800 TGs predicted by wScReNI among TGs detected by ChIP-seq for cell type-specific TFs. Targets of EBF1 and FOXP3 were detected by ChIP-seq in memory B cells and Treg cells, respectively. **B**. Precision and recall of cell type-specific regulatory networks indirectly inferred by LINGER and wScReNI across each cell type in PBMCs, utilizing various top numbers of regulation pairs. ChIP-seq, chromatin immunoprecipitation sequencing.

To validate the performance of ScReNI in inferring cell-specific regulatory networks at the cell type level, we examined whether the top regulation pairs predicted by ScReNI exhibited greater overlap with the regulatory networks detected by ChIP-seq for cell type-specific TFs. By analyzing cell type specificity of TF regulatory weights based on the cell-specific regulatory networks in PBMCs inferred by wScReNI, we found that EBF1 and FOXP3 were significantly specific to memory B cells and Treg cells, respectively (Figure S3). In the ChIP-Atlas database, 1619 TGs for EBF1 in memory B cells and 755 for FOXP3 in Treg cells were detected by ChIP-seq [34]. We overlapped the top 300, 500, and 800 TGs predicted by wScReNI and TGs detected by ChIP-seq separately for EBF1 and FOXP3. The results showed that TGs of EBF1 detected by ChIP-seq were only inferred by wScReNI in memory B cells rather than other cell types (**Figure 5**A). Moreover, a higher proportion of the top 300 TGs for EBF1 in memory B cells was detected by ChIP-seq than that of the top 500 and 800 TGs predicted by wScReNI. Similar results were observed for FOXP3. These results indicated that ScReNI reliably inferred cell-specific regulatory networks, given that the top regulation pairs predicted by ScReNI exhibited greater overlap with the regulatory networks detected by ChIP-seq at the cell type level.

To assess performance differences when analyzing paired *versus* unpaired scRNA-seq and scATAC-seq data using ScReNI, we evaluated its ability to predict cell-specific regulatory networks with scRNA-seq data alone, as well as paired and unpaired scRNA-seq and scATAC-seq data. Utilizing the paired scRNA-seq and scATAC-seq data from PBMCs, we generated and then processed unpaired data following the unpaired data processing protocol in ScReNI. The benchmark focused on the top 500 highly variable genes and the top 10,000 highly variable peaks. The results demonstrated that ScReNI consistently achieved the best performance with paired scRNA-seq and scATAC-seq data, followed by unpaired data, and then scRNA-seq data alone (Figure S4).

### ScReNI improves the inference of cell type-specific regulatory networks

For wScReNI, cell type-specific regulatory networks can be inferred either by averaging cell-specific regulatory networks within the same cell type (indirect approach) or directly using single-cell sequencing data from the same cell type as inputs of wScReNI (direct approach, see Method). We found that the precision and recall were higher for networks inferred by wScReNI using the indirect approach than those obtained with the direct approach (Figure S5). This suggests that ScReNI could provide finer regulatory insights by inferring cell-specific regulatory networks compared to cell type-specific regulatory networks.

When comparing wScReNI with other methods in cell type-specific regulatory network inference, we observed the variability of network types inferred by different methods. For example, LINGER inferred *trans*-regulation between TFs and TGs, whereas DeepTFni focused on regulatory networks between TFs. To ensure a fair assessment, we performed cell type-specific comparisons between wScReNI and each of LINGER, DeepTFni, STREAM, and scMTNI. Using different top regulation pairs, wScReNI consistently showed the highest precision and recall for top 50 and 80 regulation pairs, although the precision and recall of LINGER were slightly higher when regulation pairs exceeded 100 (Figure 5B; Table S4). Notably, wScReNI consistently outperformed DeepTFni, STREAM, and scMTNI across almost all cell type-specific regulatory networks (Figure S6).

### ScReNI shows superior runtime and memory usage

To compare the computational efficiency of ScReNI with other algorithms, we calculated the runtime and memory usage. We inferred cell-specific regulatory networks using the PBMC data comprising 400 cells, utilizing the top 500 highly variable genes and the top 10,000 highly variable peaks. Runtime and memory usage were compared across different methods (Table S5). We found that all methods had comparable memory usage. However, there was a notable variation in computational time. Specifically, two deep learning-based methods scGeneRAI and LINGER required 1534.5 min and 2756.2 min, respectively. CeSpGRN needed 201.2 min. In contrast, the remaining methods (CSN, LIONESS, kScReNI, and wScReNI) completed their computations within 50 min. Furthermore, when we processed data with 800 and 1200 cells, CSN, kScReNI, and wScReNI continued to demonstrate superior computational efficiency (Table S5). We also observed that the runtime and memory usage of ScReNI increased nearly linearly with the number of cells.

### ScReNI is applied to identify cell-enriched regulators

Using ScReNI, we sought to identify key regulators enriched in each cell-specific regulatory network, represented as cell-enriched regulators. To achieve this enrichment analysis, we performed a two-step analytical process inspired by IReNA [12]. The first step is the division of all genes in cell-specific regulatory networks into different modules by applying K-means clustering to gene expression profiles across all cells. After genes in the cell-specific regulatory network were modularized, a hypergeometric test was performed to identify which factors significantly regulate each module of genes.

**Figure 6:**
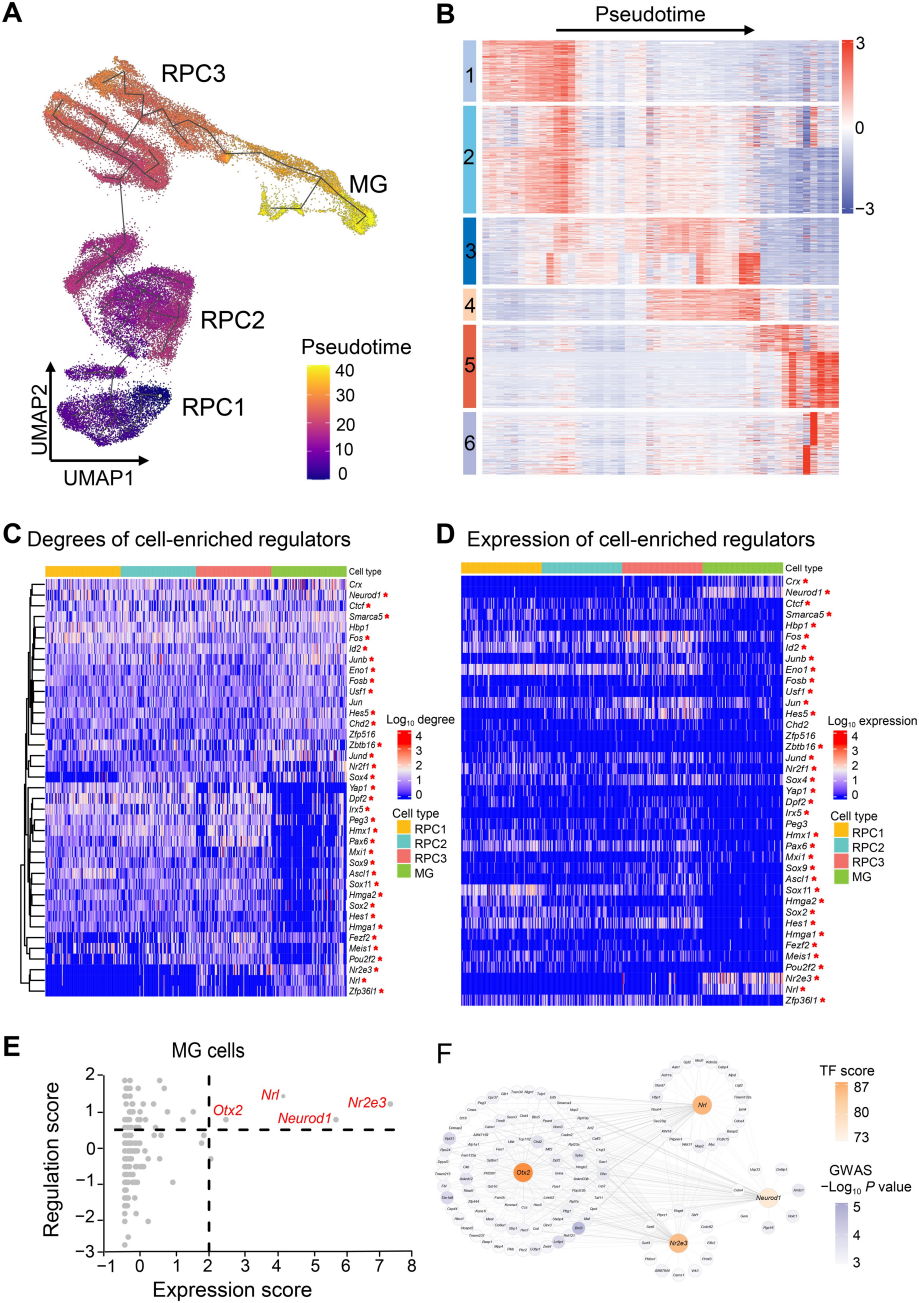
Identification of cell-enriched regulators during retinal development **A**. Pseudotime of single cells calculated by Monocle 3. **B**. Heatmap of genes divided by K-means. The color bars on the left represent six modules. **C**. Degrees of cell-enriched regulators. **D**. Expression patterns of cell-enriched regulators. *P* < 0.05 among four cell types was marked with a red star. **E**. Comparison of regulation and expression for TFs in MGs. Four key TF genes were labeled in red. **F**. Regulatory networks of four key TF genes. The color of TF genes represents trait-related score. The color of TGs indicates the significance of gene-associated SNPs in GWAS. SNP, single nucleotide polymorphism; GWAS, genome-wide association study.

Cell-specific regulatory networks from retinal development inferred by wScReNI were utilized to illustrate the identification of cell-enriched regulators. To obtain reliable gene modules, we generated the smoothed gene expression profiles according to the pseudotime of single cells. The pseudotime of single cells was calculated based on the cell trajectory inferred by Monocle 3 [32]. We observed that the pseudotime was increased by the order of RPC1, RPC2, RPC3 and MG (**Figure 6**A). Based on the smoothed gene expression profiles, we clustered the top 2000 highly variable genes into six distinct modules (Figure 6B). Each gene module was associated with specific biological functions (Figure S7A). Specifically, the first module was related to organ development and cell proliferation. The second and third modules were involved in ribosome biogenesis and chromosome segregation, respectively. The genes in the other three modules were separately enriched in gliogenesis, visual perception, and synapse assembly. These enriched functions were closely associated with retinal development.

ScReNI successfully identified cell-enriched regulators for each modularized cell-specific regulatory network. For each cell type, we calculated the frequency of enriched regulators across all 100 cells and ranked these enriched regulators according to their frequency (Table S6). By comparing the top 30 cell-enriched regulators for each cell type, we identified 39 cell-enriched regulators. Performing clustering based on the network outdegrees of these 39 cell-enriched regulators from four cell types, we identified cell type-specific regulators and shared regulators (Figure 6C). Notably, 35 of 39 regulators showed statistically significant differences among four cell types (RPC1, RPC2, RPC3, and MG) in terms of network degree, as indicated by red asterisks in Figure 6C. Interestingly, 33 of these 35 regulators showed significant differences among four cell types, not only in network degree, but also in expression levels (Figure 6D). In particular, the cell-enriched regulators *Nr2e3* and *Nrl* had significantly higher network degrees in MGs than in RPCs, with their expression levels also upregulated in MGs (Figure 6C and D). Spearman correlation coefficients between network degrees and expression levels of the regulators *Nr2e3* and *Nrl* were 0.39 and 0.40, respectively. According to the scATAC-seq data, the regulatory activities of *Nr2e3* and *Nrl* were significantly higher in MGs than in RPCs (Figure S7B). Another regulator, *Zfp36l1*, had a significantly higher network degree in MGs than in RPCs, but its expression level was downregulated in MGs (Figure 6C and D). The Spearman correlation coefficient between network degree and expression level of the regulator *Zfp36l1* was −0.28. This observation could be due to the fact that *Zfp36l1* was negatively regulated by other TFs in MGs but not in RPCs (Figure S7C). We also found that *Yap1*, *Dpf2*, *Hmx1*, *Pax6*, *Hmga2*, *Sox2*, *Hes1*, and *Meis1* had higher network degrees in RPCs than in MGs. Accordingly, the expression levels of these factors were upregulated in RPCs. These factors showed positive correlations between network degrees and expression levels. Additionally, two regulators *Chd2* and *Peg3* showed network-based cell type specificity, but did not exhibit cell type specificity in terms of gene expression and regulatory activity.

### ScReNI is utilized to reveal the regulatory landscape of GWAS traits

To identify cell types, key TFs, and regulatory networks associated with GWAS traits, we integrated GWAS summary statistics with cell type-specific regulatory networks using the methods described by Yuan and Duren [21]. In the study of age-related macular degeneration (AMD), we compiled 1400 risk loci from a GWAS meta-analysis, sourced from the EMBL’s European Bioinformatics Institute (EMBL-EBI) GWAS Catalog, which includes data from 77 studies.

From this dataset, we identified 236 trait-related genes and selected the 500 top-ranked genes based on *trans*-regulation to serve as TGs for each TF. We then counted the number of overlapping genes between the trait-related genes and the TGs. The most relevant cell type was MG, which was assessed using a *t*-test comparing the number of overlapping genes between the 20 top-expressed TFs and 20 randomly chosen TFs. These results are consistent with previous studies that link MG to the pathogenesis of AMD [35].

Next, we identified four key genes encoding TFs (*Otx2*, *Nrl*, *Neurod1*, and *Nr2e3*) by summing their mRNA expression levels and trait regulation scores (Figure 6E). All these TFs are related to retinal degeneration according to the literature [36–39]. Finally, we extracted the regulatory network of the four key TFs from the MG regulatory network for AMD (Figure 6F).

## Discussion

Single cells exhibit heterogeneous gene expression profiles and chromatin accessibility, measurable separately via scRNA-seq and scATAC-seq. Consequently, each cell possesses a unique gene regulatory network. However, limited methods exist for inferring cell-specific regulatory networks, particularly when dealing with unpaired scRNA-seq and scATAC-seq data. Herein, we developed ScReNI, a computational method designed to infer cell-specific regulatory networks by integrating scRNA-seq and scATAC-seq data. Using regulatory relationships detected by ChIP-seq data as the ground truth, we demonstrated that ScReNI outperformed existing methods in predicting cell-specific networks. Moreover, single-cell clustering analysis based on the gene degrees of cell-specific networks also indicated that ScReNI excelled in distinguishing different cell types. Lastly, a unique function in ScReNI was developed to identify cell-enriched regulators, offering new insights into cell-specific regulatory mechanisms.

In current single-cell genomics studies, the integration of scRNA-seq and scATAC-seq data is a challenge due to the technical batch effect. Existing methods for data integration include anchoring methods, transfer learning methods, manifold alignment, matrix factorization, and neural network methods [27,40–43]. The advantage of data integration is that it improves the accuracy of identifying nearest neighbors and inferring gene regulatory relationships. ScReNI assumes that cells with similar molecular profiles share analogous gene regulatory networks. The regulatory network of a single cell is represented by the network inferred from its nearest neighbors. To identify these neighbors, ScReNI uses Seurat’s weighted-nearest neighbor analysis for paired scRNA-seq and scATAC-seq data and Seurat’s anchoring procedure followed by Harmony for unpaired scRNA-seq and scATAC-seq data. Based on random forest, we developed a new method in ScReNI to establish nonlinear relationships between gene expression and chromatin accessibility for cell-specific regulatory network inference. This is different from the existing methods such as CeSpGRN which incorporates prior knowledge from one omics to another omics data [20]. Additionally, the random forest model incorporated the association between TGs, regulatory factors, and peaks based on genomic proximity and motif matching, which enhances the accuracy of regulatory network inference. By integrating single-cell multi-omics data within a single model, ScReNI elucidates a superior regulatory landscape at the single-cell level. Future research will include a more comprehensive and theoretically grounded investigation of different approaches to elucidate their comparative effectiveness in inferring regulatory networks.

Despite existing methods, such as SCENIC+ and IReNA [12,13], showing promising performance in determining key regulators in cell type-specific networks, it remains a challenge to identify statistically enriched regulators at the single-cell level. Here, we demonstrated that ScReNI could enrich regulators within each cell-specific network. For instance, ScReNI effectively pinpointed TFs that acted as regulatory factors for specific cell types by identifying cell-enriched regulators. These TFs, including those encoded by *Nrl* and *Nr2e3*, are notable not only for their regulatory activity but also for their distinctive expression profiles. By identifying cell-enriched regulators, we can unravel the intricate regulatory relationships between TFs and TGs, facilitating a more targeted investigation of the underlying regulatory biology.

The cell-enriched regulators *Nrl* and *Nr2e3* had higher network degrees in MGs than in RPCs, with their mRNA expression levels upregulated in MGs and downregulated in RPCs. This is consistent with the previous report that *Nrl* could activate *Nr2e3* to suppress photoreceptor development [44]. In addition, the enriched regulators *Yap1*, *Dpf2*, *Hmx1*, *Pax6*, *Hmga2*, *Sox2*, *Hes1*, and *Meis1*, whose network degrees were higher in RPCs than in MGs, showed a similar pattern of upregulation in RPCs and downregulation in MGs in terms of their expression levels. All these regulators, except *Dpf2*, have been reported to regulate RPCs [45–48]. Notably, *Chd2* and *Peg3* showed a significant difference in network degree among four cell types, but not in expression level or regulatory activity, consistent with the previous concept of “dark” genes [17]. The study demonstrated that ScReNI can reliably infer cell-specific regulatory networks and efficiently identify cell-enriched regulators, which has important implications for the regulatory study of diverse biological processes, such as tissue development.

## Code availability

The R package of ScReNI is available on GitHub (https://github.com/Xuxl2020/ScReNI). And it has also been submitted to BioCode at the National Genomics Data Center (NGDC), China National Center for Bioinformation (CNCB) (BioCode: BT007773), which is publicly accessible at https://ngdc.cncb.ac.cn/biocode/tool/7773.

## CRediT author statement

**Xueli Xu:** Conceptualization, Methodology, Formal analysis, Investigation, Software, Writing – original draft, Writing – review & editing. **Yanran Liang:** Validation, Resources. **Miaoxiu Tang:** Validation, Writing – review & editing. **Jiongliang Wang:** Validation. **Xi Wang:** Writing – review & editing. **Yixue Li:** Writing – review & editing, Supervision. **Jie Wang:** Conceptualization, Writing – review & editing, Supervision, Project administration, Funding acquisition. All authors have read and approved the final manuscript.

## Competing interests

The authors have declared no competing interests.

## Supplementary Material

qzaf060_Supplementary_Data
