# Supplementary material for: ScReNI: Single-cell Regulatory Network Inference Through Integrating scRNA-seq and scATAC-seq Data
Source: Genomics Proteomics Bioinformatics. 2025 Jul 1;23(4):qzaf060. doi: 10.1093/gpbjnl/qzaf060 (PMC12646639; doi:10.1093/gpbjnl/qzaf060)
Supplement: qzaf060_Supplementary_Data [file qzaf060_supplementary_data.zip › FigS1.pdf]

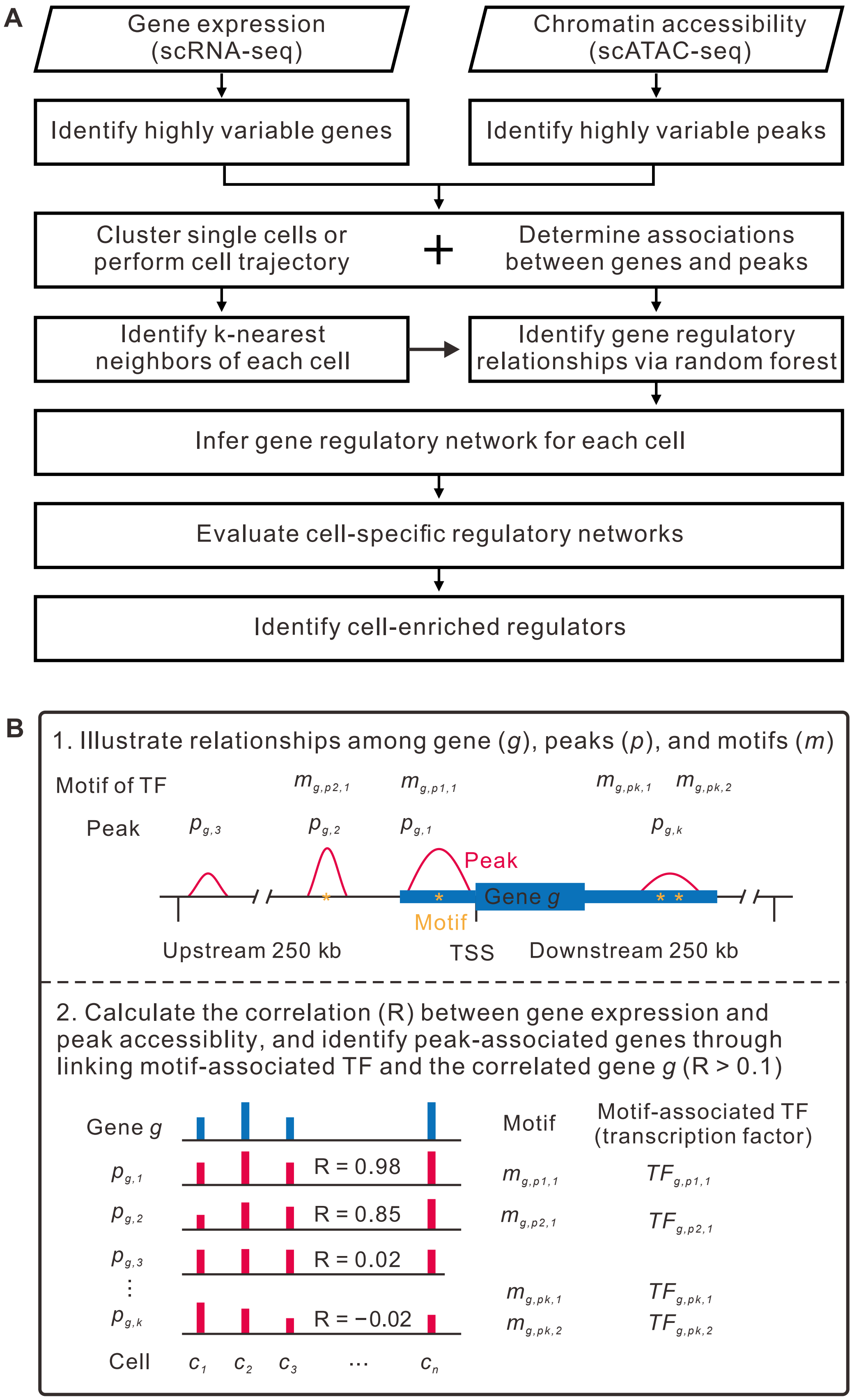
A
Gene expression (scRNA-seq)
Chromatin accessibility (scATAC-seq)
Identify highly variable genes
Identify highly variable peaks
Cluster single cells or perform cell trajectory
+
Determine associations between genes and peaks
Identify k-nearest neighbors of each cell
Identify gene regulatory relationships via random forest
Infer gene regulatory network for each cell
Evaluate cell-specific regulatory networks
Identify cell-enriched regulators
B
1. Illustrate relationships among gene (g), peaks (p), and motifs (m)
Motif of TF
$m_{g,p2,1}$ $m_{g,p1,1}$ $m_{g,pk,1}$ $m_{g,pk,2}$
Peak
$p_{g,3}$ $p_{g,2}$ $p_{g,1}$ $p_{g,k}$
Peak
Gene g
Motif
Upstream 250 kb
TSS
Downstream 250 kb
2. Calculate the correlation (R) between gene expression and peak accessiblity, and identify peak-associated genes through linking motif-associated TF and the correlated gene g (R > 0.1)
Gene g
Motif
Motif-associated TF (transcription factor)
$p_{g,1}$ R = 0.98 $m_{g,p1,1}$ $TF_{g,p1,1}$
$p_{g,2}$ R = 0.85 $m_{g,p2,1}$ $TF_{g,p2,1}$
$p_{g,3}$ R = 0.02
$p_{g,k}$ R = −0.02 $m_{g,pk,1}$ $m_{g,pk,2}$ $TF_{g,pk,1}$ $TF_{g,pk,2}$
Cell c_1 c_2 c_3 … c_n
