# Supplementary material for: ScReNI: Single-cell Regulatory Network Inference Through Integrating scRNA-seq and scATAC-seq Data
Source: Genomics Proteomics Bioinformatics. 2025 Jul 1;23(4):qzaf060. doi: 10.1093/gpbjnl/qzaf060 (PMC12646639; doi:10.1093/gpbjnl/qzaf060)
Supplement: qzaf060_Supplementary_Data [file qzaf060_supplementary_data.zip › FigS2.pdf]

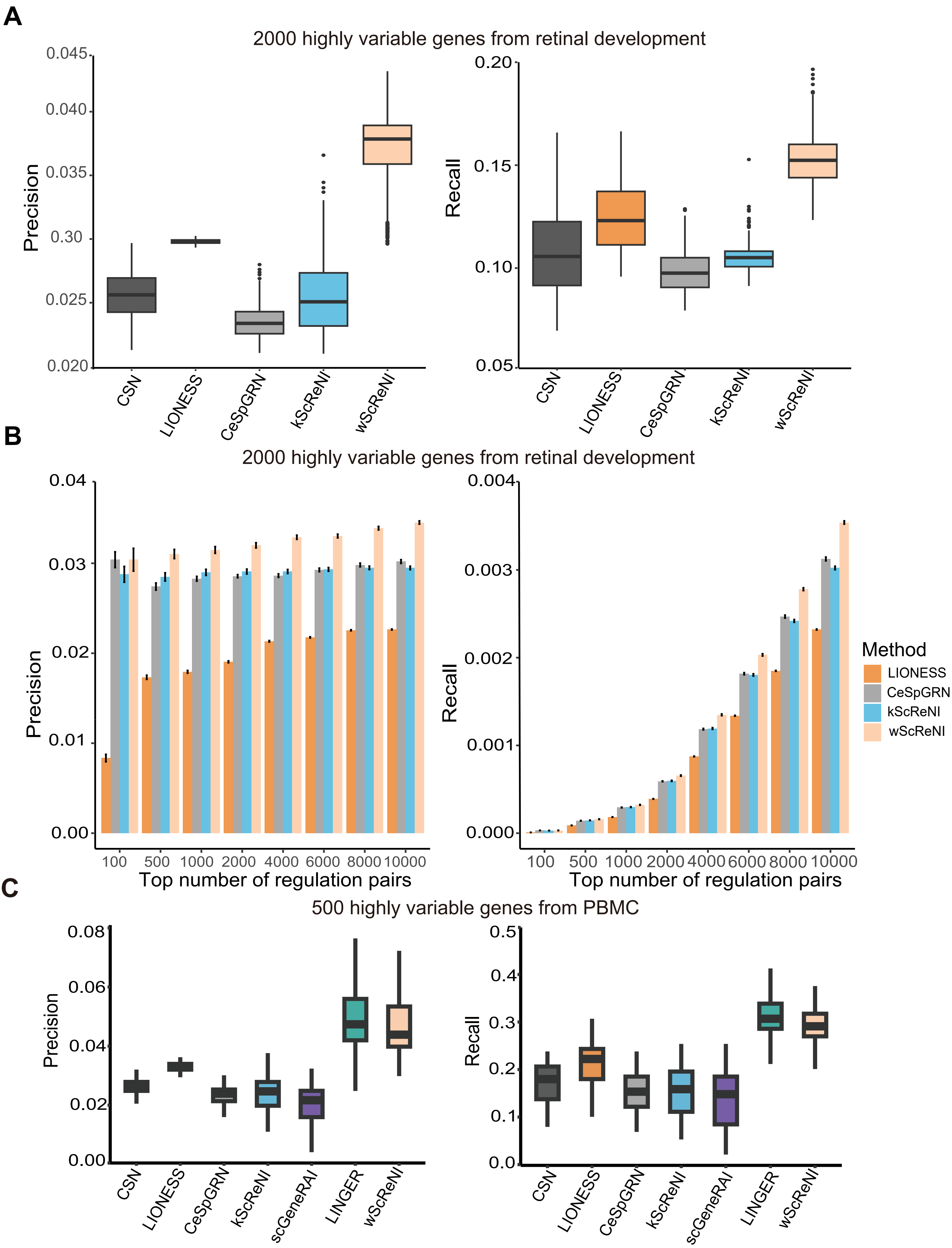

A
2000 highly variable genes from retinal development
Precision
0.045
0.040
0.035
0.30
0.025
0.020
Recall
0.20
0.15
0.10
0.05
CSN
LIONESS
CeSpGRN
kScReNI
wScReNI
B
2000 highly variable genes from retinal development
Precision
0.04
0.03
0.02
0.01
0.00
Recall
0.004
0.003
0.002
0.001
0.000
100
500
1000
2000
4000
6000
8000
10000
Top number of regulation pairs
Method
LIONESS
CeSpGRN
kScReNI
wScReNI
C
500 highly variable genes from PBMC
Precision
0.08
0.06
0.04
0.02
0.00
Recall
0.5
0.4
0.3
0.2
0.1
0.0
CSN
LIONESS
CeSpGRN
kScReNI
scGeneRAI
LINGER
wScReNI
