# Supplementary material for: ScReNI: Single-cell Regulatory Network Inference Through Integrating scRNA-seq and scATAC-seq Data
Source: Genomics Proteomics Bioinformatics. 2025 Jul 1;23(4):qzaf060. doi: 10.1093/gpbjnl/qzaf060 (PMC12646639; doi:10.1093/gpbjnl/qzaf060)
Supplement: qzaf060_Supplementary_Data [file qzaf060_supplementary_data.zip › Supplementary material captions.docx]

**Supplementary material**

**Figure S1 Illustration of single-cell network inference methods**

**A.** Workflow of ScReNI. **B.** Schematics of the association of gene expression from scRNA-seq and peak accessibility from scATAC-seq. TSS, transcription start site.

**Figure S2 Performances of cell-specific network inference methods from retinal development and PBMC**

**A.** Precision and recall of single-cell regulatory relationships using the same number of regulation pairs in CSN. **B.** Precision and recall of single-cell regulatory relationships using different top numbers of regulation pairs. **C.** Precision and recall of single-cell regulatory relationships using the same number of regulation pairs in CSN.

**Figure S3 Regulatory weights of transcription factors *EBF1* and *FOXP3* inferred by wScReNI across cell types in PBMC**

**Figure S4 Network analysis of scRNA-seq data, unpaired, and paired scRNA-seq and scATAC-seq data in PBMC**

**A.** Precision and recall of single-cell regulatory relationships using different top numbers of regulation pairs. **B.** Comparison of network-based cell clustering.

**Figure S5 Precision and recall of cell type-specific regulatory networks inferred by wScReNI using indirect and direct approaches in PBMC**

**Figure S6 Benchmarking the performance of cell type-specific regulatory network inference**

**A.** Precision and recall of TF regulatory networks inferred by DeepTFni and wScReNI across each cell type in PBMC. **B.** Precision and recall of enhancer-driven gene regulatory networks inferred by STREAM and wScReNI across each cell type in PBMC. **C.** Precision and recall of regulatory network inferred by scMTNI and wScReNI across each cell type from retinal development. scMTNI, single-cell multi-task network inference; STREAM, single-cell trajectory reconstruction, exploration and mapping of single-cell data.

**Figure S7 Enrichment analysis of the top highly variable genes in retinal development**

**A.** Enriched functions for genes across six modules. **B.** Activities of cell-enriched regulators. *P* < 0.05 among four cell types was marked with a red star. **C.** Average number of genes regulating *Zfp36l1* negatively and positively in MGs and RPCs.

**Table S1 Comparison of network-based cell clustering in retinal development**

**Table S2 Comparison of network-based cell clustering in PBMC**

**Table S3 Comparison of cell clustering based on cell-specific regulatory networks inferred by LINGER and wScReNI in PBMC**

**Table S4 Precision and recall of averaging cell-specific networks for LINGER and wScReNI using different top numbers of regulation pairs in PBMC**

**Table S5 Runtime and memory usage for ScReNI and other methods**

**Table S6 The list of frequencies of cell-enriched regulators in MG, RPC1, RPC2, and RPC3**
