# Supplementary figures and images for: ScReNI: Single-cell Regulatory Network Inference Through Integrating scRNA-seq and scATAC-seq Data
Source: Genomics Proteomics Bioinformatics. 2025 Jul 1;23(4):qzaf060. doi: 10.1093/gpbjnl/qzaf060 (PMC12646639; doi:10.1093/gpbjnl/qzaf060)

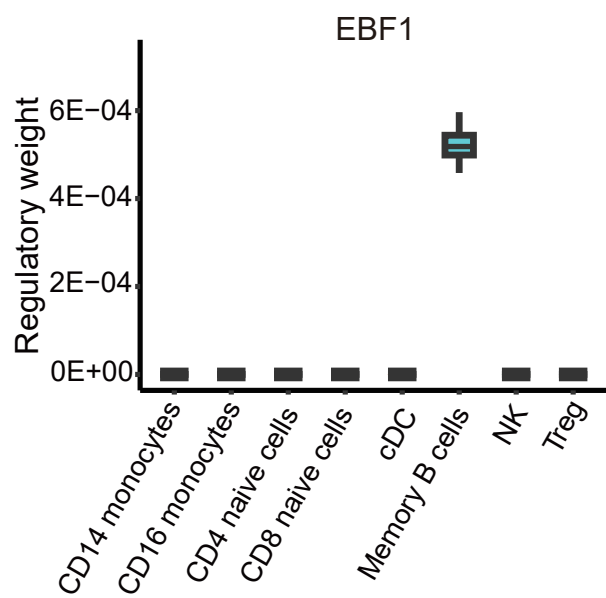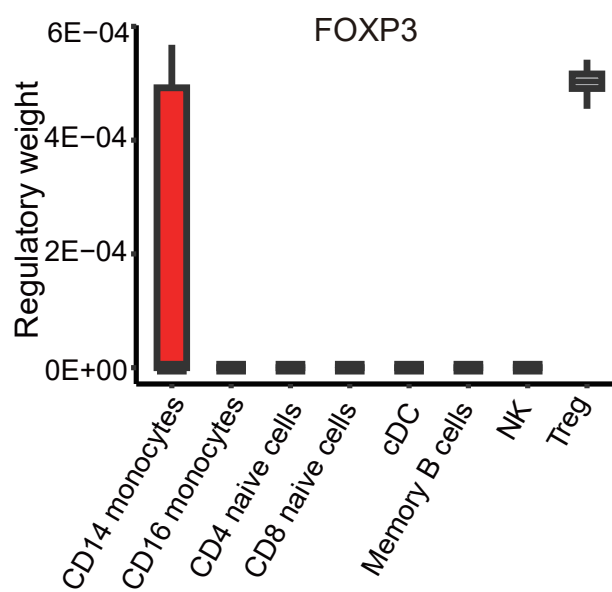

Supplement: qzaf060_Supplementary_Data [file qzaf060_supplementary_data.zip › FigS3.pdf]

**A**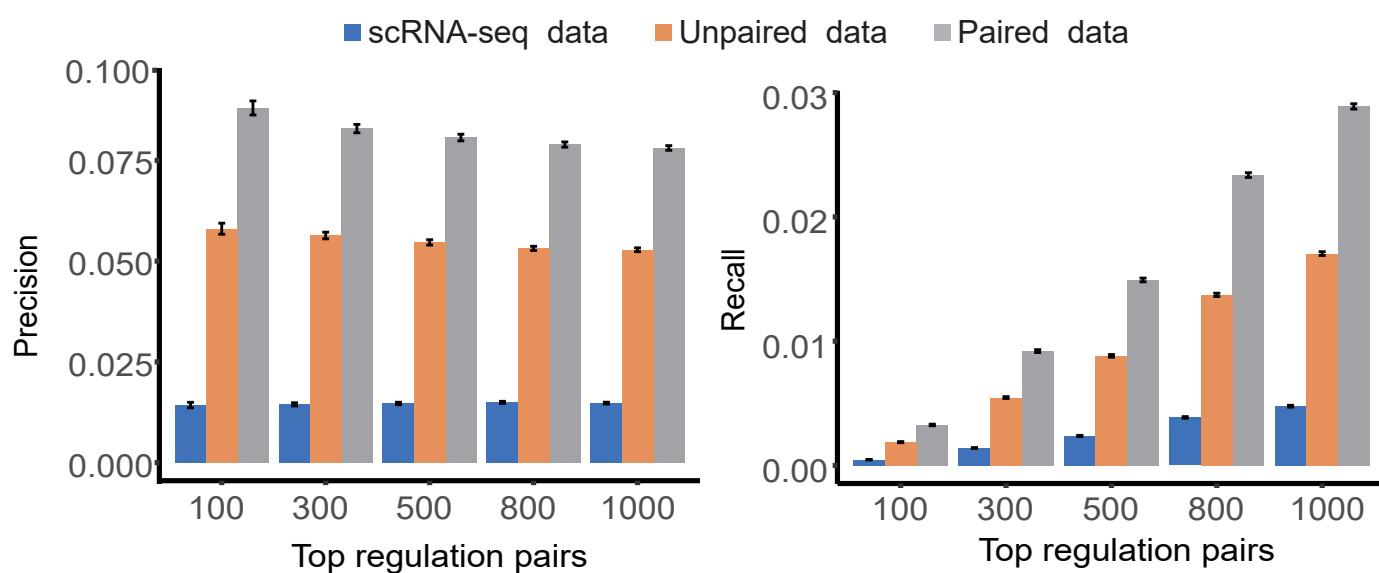**B**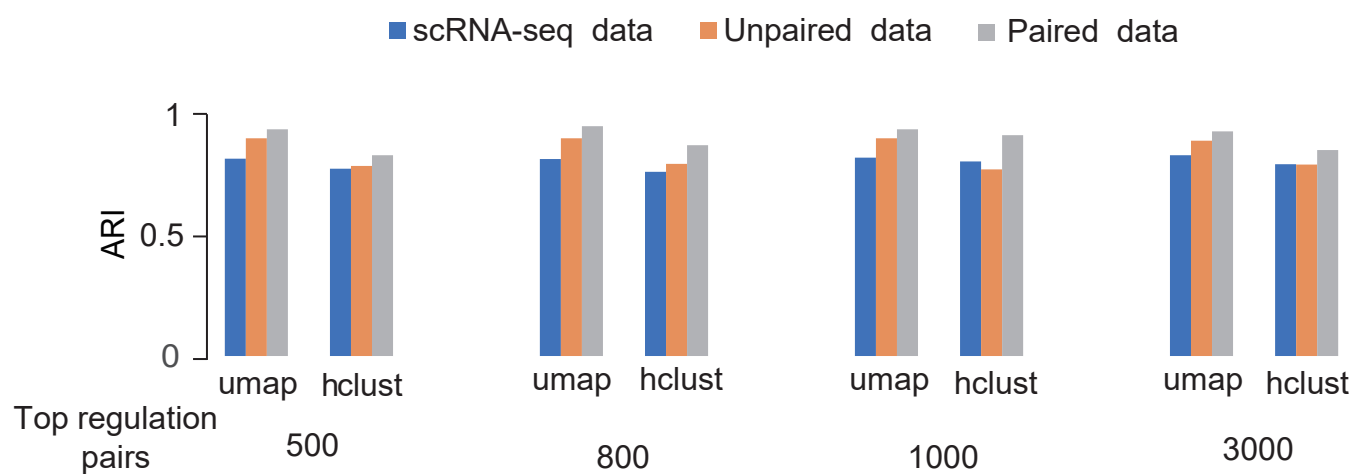

Supplement: qzaf060_Supplementary_Data [file qzaf060_supplementary_data.zip › FigS4.pdf]

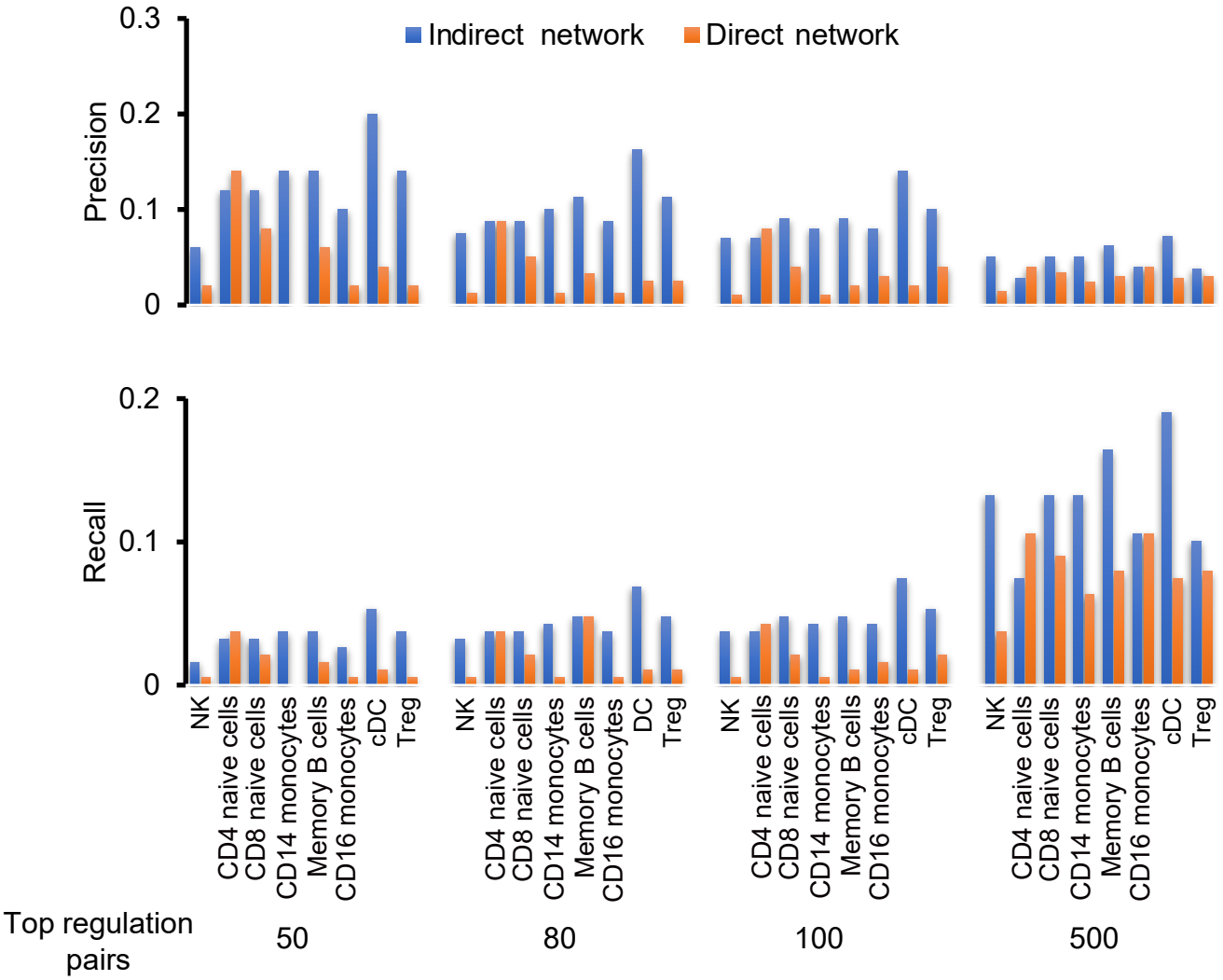

Supplement: qzaf060_Supplementary_Data [file qzaf060_supplementary_data.zip › FigS5.pdf]

**A**

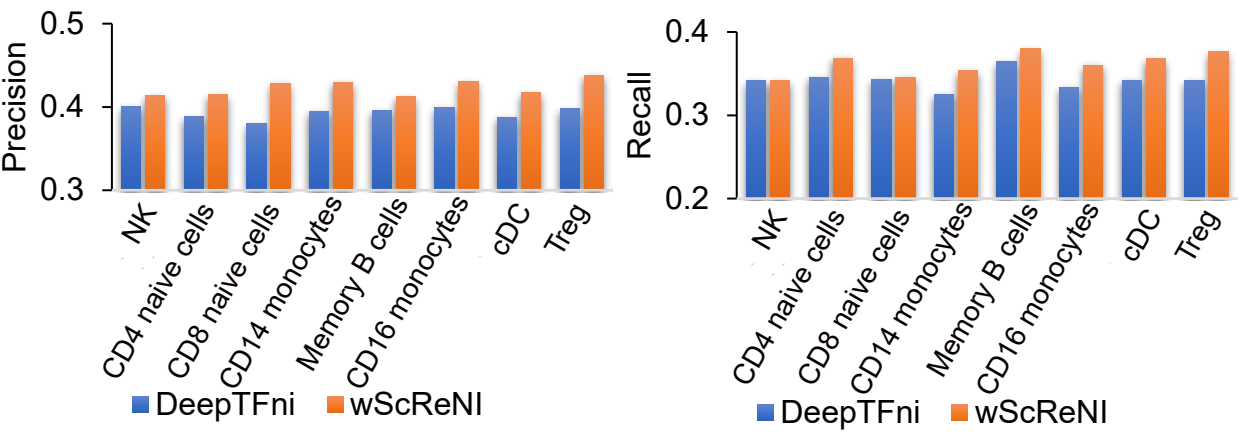

**B**

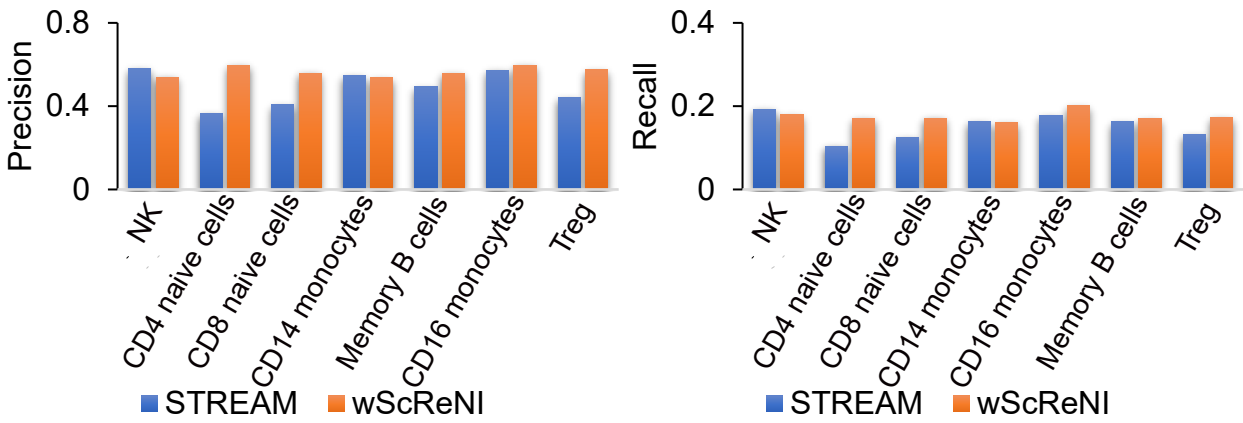

**C**

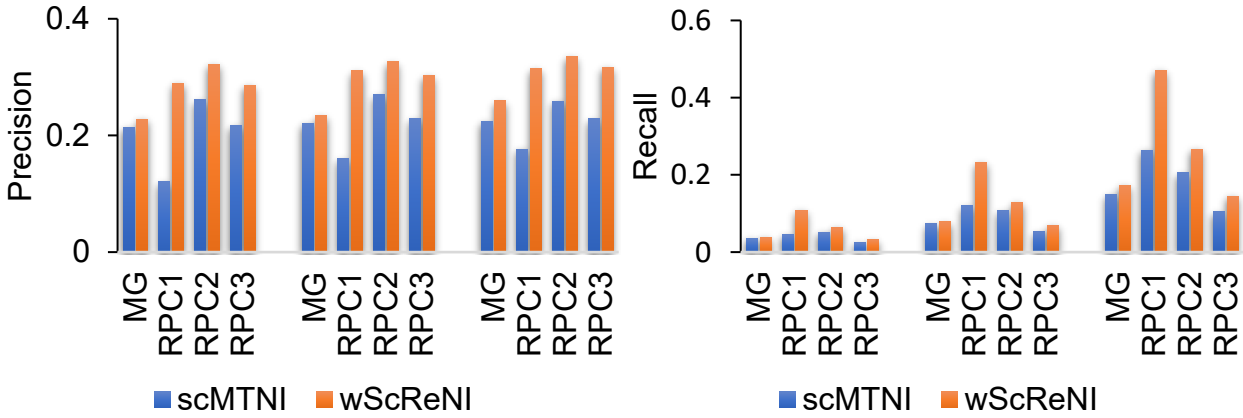

Supplement: qzaf060_Supplementary_Data [file qzaf060_supplementary_data.zip › FigS6.pdf]

**A**

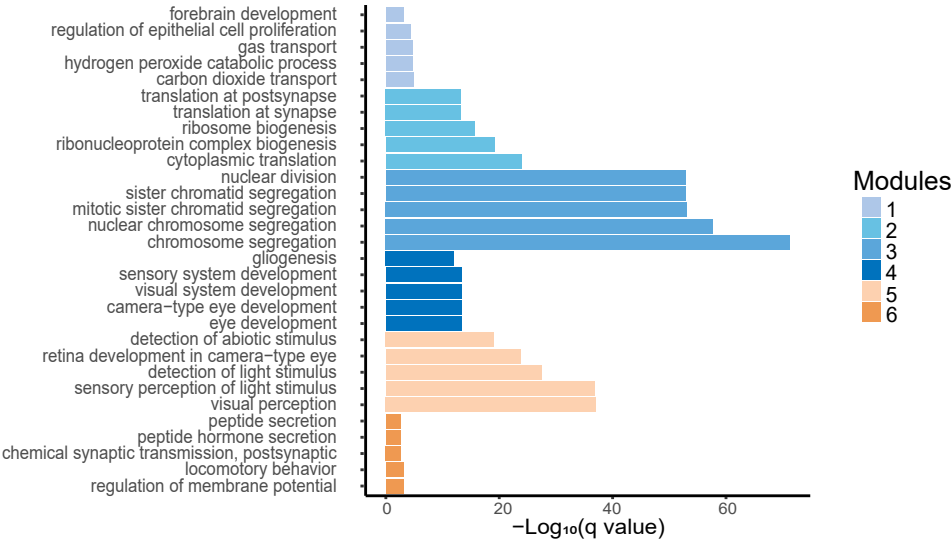

**B**

Regulatory activities of cell-enriched regulators

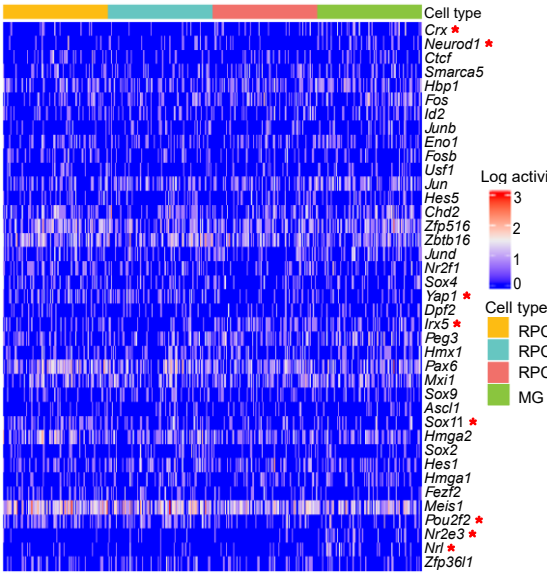

**C**

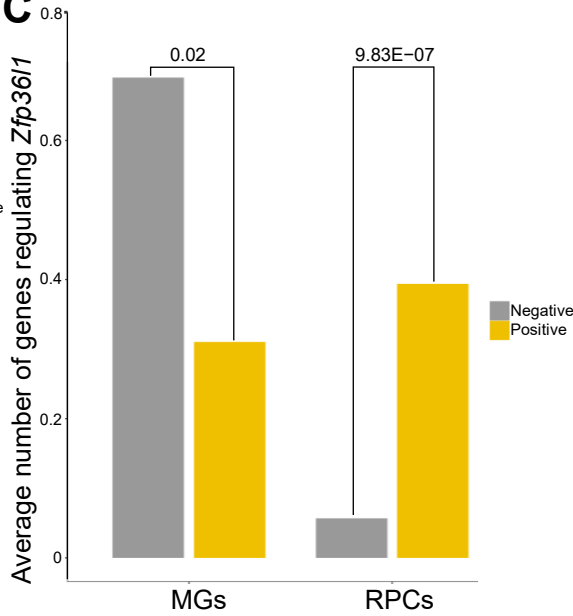

Supplement: qzaf060_Supplementary_Data [file qzaf060_supplementary_data.zip › FigS7.pdf]
